# Diversity and Evolution of Type IV pili Systems in Archaea

**DOI:** 10.3389/fmicb.2016.00667

**Published:** 2016-05-06

**Authors:** Kira S. Makarova, Eugene V. Koonin, Sonja-Verena Albers

**Affiliations:** ^1^National Center for Biotechnology Information, National Library of Medicine – National Institutes of HealthBethesda, MD, USA; ^2^Molecular Biology of Archaea, Institute of Biology II, University of FreiburgFreiburg, Germany

**Keywords:** type IV pili, archaea, evolution, comparative genomics, secretion ATPase

## Abstract

Many surface structures in archaea including various types of pili and the archaellum (archaeal flagellum) are homologous to bacterial type IV pili systems (T4P). The T4P consist of multiple proteins, often with poorly conserved sequences, complicating their identification in sequenced genomes. Here we report a comprehensive census of T4P encoded in archaeal genomes using sensitive methods for protein sequence comparison. This analysis confidently identifies as T4P components about 5000 archaeal gene products, 56% of which are currently annotated as hypothetical in public databases. Combining results of this analysis with a comprehensive comparison of genomic neighborhoods of the T4P, we present models of organization of 10 most abundant variants of archaeal T4P. In addition to the differentiation between major and minor pilins, these models include extra components, such as S-layer proteins, adhesins and other membrane and intracellular proteins. For most of these systems, dedicated major pilin families are identified including numerous stand alone major pilin genes of the PilA family. Evidence is presented that secretion ATPases of the T4P and cognate TadC proteins can interact with different pilin sets. Modular evolution of T4P results in combinatorial variability of these systems. Potential regulatory or modulating proteins for the T4P are identified including KaiC family ATPases, vWA domain-containing proteins and the associated MoxR/GvpN ATPase, TFIIB homologs and multiple unrelated transcription regulators some of which are associated specific T4P. Phylogenomic analysis suggests that at least one T4P system was present in the last common ancestor of the extant archaea. Multiple cases of horizontal transfer and lineage-specific duplication of T4P loci were detected. Generally, the T4P of the archaeal TACK superphylum are more diverse and evolve notably faster than those of euryarchaea. The abundance and enormous diversity of T4P in hyperthermophilic archaea present a major enigma. Apparently, fundamental aspects of the biology of hyperthermophiles remain to be elucidated.

## Introduction

Most bacteria and archaea produce numerous morphologically diverse cell surface structures that enable cell motility, cell–cell interactions and surface attachment (Albers and Meyer, 2011; Berry and Pelicic, 2015; Costa et al., 2015; Pohlschroder and Esquivel, 2015). The assembly of most of such structures requires energy-dependent secretion of the building blocks. In accord with this functional linkage, the genes for secretion apparatus and extracellular components often form operons and are jointly referred to as secretion or pili assembly systems. The majority of surface structures in archaea that have been experimentally studied to date resemble bacterial type IV pili (T4P) and type II secretion systems (T2SS) (Pohlschroder et al., 2011). These systems are very abundant in bacteria (Nivaskumar and Francetic, 2014; Berry and Pelicic, 2015). In contrast to T4P, which assemble and disassemble the pilus filament to adhere to surfaces and promote twitching motility or adherence, T2SS mediate secretion of a substrate protein across the outer membrane in Gram-negative bacteria. These systems encompass at least four main structural and functional components, namely an assembly ATPase, a membrane platform, pilins, the building blocks of surface structures, and a prepilin peptidase (Tomich et al., 2007; Ng et al., 2008; Ayers et al., 2010; Lassak et al., 2012). Phylogenetic analysis of the secretion ATPases has shown that most archaeal proteins in this family belong to a monophyletic group within a major branch, which encompasses ATPases of Type IV secretion systems (T4SS) (Planet et al., 2001; Tomich et al., 2007). However, some other features of archaeal proteins associated with these ATPases resemble components of bacterial T4P or T2SS. Specifically, the prepilin processing is catalyzed by a dedicated peptidase of the PilD family which is the hallmark of bacterial T4P and T2SS (Strom et al., 1993). The PilD peptidase cleaves before the hydrophobic part of the tripartite signal peptide forming an α-helix, which is essential for the formation of type IV pilus structure, whereas in typical secretory signal peptides, this part of the signal peptide is fully removed (Strom et al., 1993). Archaeal type IV prepilins and archaellins are processed by an archaeal homolog of PilD, known as PibD or FlaK, and are assembled in the same manner as bacterial T4P and T2SS, suggesting a common origin of these components (Bardy and Jarrell, 2002; Albers et al., 2003). Therefore, the archaeal secretion-pili systems appear to be chimeric, with the ATPases and a membrane platform protein being more closely related to T4SS group (including Tad-like systems that are referred as T4P), whereas the pilins and pre-pilin peptidases derived from T2SS and related T4P.

Recently, several archaeal systems of this class have been experimentally characterized including the archaellum (Jarrell and Albers, 2012), UV-induced pili (Frols et al., 2008; van Wolferen et al., 2013), and the bindosome (Zolghadr et al., 2011) in *Sulfolobus* species, and a pili system in *Methanococcus maripaludis* as well as some other class I methanogens, Thermococci, *Halobacterium salinarum*, and *Haloferax volcanii* (Albers and Meyer, 2011; Esquivel et al., 2013; Nair et al., 2013; Losensky et al., 2014). With exception of the specialized archaellum, all these systems are different variants of pili and thus are hereinafter will be referred to as T4P. Despite the recent progress in the experimental characterization of the archaeal pili systems (Pohlschroder and Esquivel, 2015), the majority of T4P in archaea remain uncharacterized either experimentally or in terms of genetic organization and molecular componentry.

In the course of a recent analysis of the genomic “dark matter” in archaea, we have identified several loci in a variety of archaeal genomes that encode homologs of secretion ATPases together with many other proteins without identifiable similarity to known components of either T4P or other presently identified classes of secretion systems (Makarova et al., 2014). This finding prompted us to perform a comprehensive analysis of the gene composition and evolution of T4P and related membrane systems in archaea.

## Materials and Methods

### Genome Sequences and Sequence Analysis

Genomes of 168 archaea were downloaded from the NCBI FTP site^1^. Proteins were assigned to arCOGs as described previously (Wolf et al., 2012). Phyletic patterns of arCOGs and their annotations were obtained from the latest release of the arCOG database (Makarova et al., 2015). Sequence similarity was analyzed using PSI-BLAST (Altschul et al., 1997) and HHpred (Soding et al., 2005) programs. Transmembrane segments in protein sequences were predicted using the TMHMM v. 2.0c program with default parameters (Krogh et al., 2001). Signal peptides were predicted using the SignalP v. 4.1c program; the union of three predictions (Gram-negative, Gram-positive and eukaryotic models) was used (Petersen et al., 2011). Flafind 1.2 server was used to identify proteins matching archaeal archaellin signature (Esquivel et al., 2013). Multiple sequence alignments were constructed using MUSCLE (Edgar, 2004). For phylogenetic reconstruction sites with the gap character fraction >0.5 and homogeneity <0.1 (Yutin et al., 2008) were removed. The FastTree program (Price et al., 2010) with WAG evolutionary model and discrete gamma model with 20 rate categories was used for phylogenetic tree reconstruction^2^.

## Results

### Phylogenetic Analysis of Secretion ATPases in Archaea

The secretion ATPase (often referred as VirB11) family that contains various proteins, such as CpaF, TadA, GspE, PulE, PilT, and many others (Ayers et al., 2010), is the most highly conserved and common component of T2SS, T4P, and T4SS systems and thus is considered as a marker of secretion and assembly systems. Based on multiple shared sequence and structural features, these ATPases have been identified as a distinct clade within the FtsK-HerA superfamily of pumping ATPases. In the evolutionary tree of this superfamily, the secretion ATPases (typified by MJ1533 protein from *Methanocaldococcus jannaschii*) form a clade with the pilus retraction ATPase PilT and an archaea-specific family in which ATPase domain is fused to a PIN and KH domains (predicted RNAse and RNA-binding domain, respectively) (Iyer et al., 2004). According to the arCOG database (Makarova et al., 2015) archaea do not encode members of the PilT ATPase family (COG2805). The MJ1533-like genes have never been identified in genomic contexts characteristic of secretion systems and can be predicted to function in DNA or RNA repair or RNA metabolism. Archaeal secretion ATPases of the VirB11 family are monophyletic in the tree that includes T2SS or T4SS ATPases from both archaea and bacteria (Planet et al., 2001) and thus can be analyzed separately from the bacterial homologs. This group consists of 5 arCOGs (arCOG01817, arCOG01818, arCOG01819, arCOG05609, arCOG05558; 537 proteins altogether), at least one of which is present in the majority of archaea (Supplementary Table S1, phyletic patterns). The exceptions include three species of Methanosaeta and three species of Themoplasmatales, which appears consistent with the known phenotypes of these organisms: Methanosaeta species are slow growing aquatic archaea that have not been observed to interact with other organisms or to possess archaella. Themoplasmatales species Methanomassiliicoccus are not motile (Dridi et al., 2012) which is consistent at least with the absence of an archaellum. Finally, the secretion ATPases are missing in *Nitrosopumilus maritimus* SCM1 (Thaumarchaeota) (Konneke et al., 2005); the biology of these organisms is poorly understood, so the causes of this absence remain obscure. The number of T4P encoded in archaeal genomes, with the number of distinct VirB11 ATPases taken as a proxy, varies from 1 (e.g., in Methanobacterium species) to 7 (e.g., in *Vulcanisaeta distributa*). Halobacteria and Methanosarcinales generally possess more such systems than other groups of archaea (Supplementary Table S1, phyletic patterns).

**FIGURE 1:**
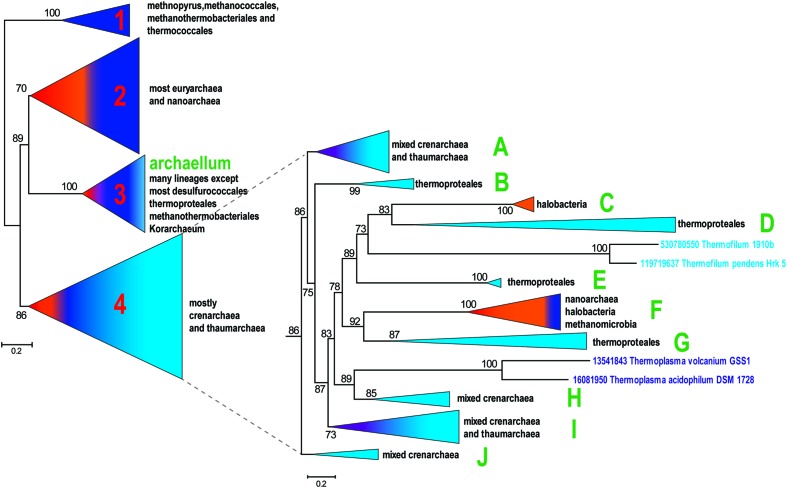
**Schematic phylogenetic tree of archaeal secretion ATPases.** The tree was reconstructed from a multiple alignment containing 356 sequences and 268 aligned positions. The bootstrap values are shown for clades with >70% support. All clades except 4 major ones are collapsed in the left panel and the topology of clade 4 is detailed in the right panel. The subclades of clade 4 that are discussed in the text are denoted A to J. Comment about the presence of major archaeal lineages is provided for each collapsed clade on the right side. Color code: Euryarchaeota, dark blue, with the exception of Halobacteria that are shown in orange; Crenarchaeota, light blue; Nanoarchaeota, red; Thaumarchaeota, Aigarchaeaota, and Korarchaeota, purple.

A representative set of proteins from arCOGs that include VirB11 family ATPases (356 proteins from 126 archaeal genomes) was selected using as a guide to build the archaeal species tree for the same organisms (Makarova et al., 2015) (Supplementary Table S2). The phylogenetic tree for this set of secretion ATPases is shown in **Figure 1** (See also Supplementary File S1). The resulting tree topology is generally consistent with phylogenetic analysis of a much smaller subset of these proteins published previously (Planet et al., 2001; Tomich et al., 2007). Clade 1 was the deepest in those trees and therefore was chosen as an outgroup (**Figure 1**). It includes only representatives of methanococci, methanobacteria, and thermococci and most likely corresponds to a distinct pili system characterized in *M. maripaludis* (Nair et al., 2013). The rest of the tree can be divided into three major clades that are largely reproducible irrespective of the set of selected organisms or the number of informative positions in the alignment (data not shown). However, the branching order depends of the sequence set and the number of positions in alignment used for tree reconstruction and therefore cannot be considered robust (data not shown). Clade 2 is mostly represented in euryarchaea, clade 3 includes archaellum-associated ATPases that are present in many diverse archaea and clade 4 includes mostly members of the TACK (thaumarchaea, aigarchaea, crenarchaea, Korarchaea) superphylum, but also a few euryarchaeotes, primarily halobacteria (**Figure 1**, Supplementary Table S3, Four major clades phyletic pattern).

Given that the branching order of clades 2, 3, and 4 is not fully reliable, the evolutionary scenario that led to the extant diversity of archaeal T4P remains unclear. Two scenarios of the archaellum origin (clade 3) are possible: (i) the archaellum antedates the LACA (last archaeal common ancestor) or (ii) the archaellum evolved within one of the major clades after the divergence of Euryarchaeota and TACK superphylum. Given the absence of the archaellum in Korarchaeon, most desulfurococcales and thermoproteales (see Supplementary Table S3), the origin of archaellum in Euryarchaeota, followed by HGT (horizontal gene transfer) to the ancestor of Sulfolobales and to several thaumarchaea appears to be the most parsimonious scenario which is also consistent with tree topology where clade 2 is the sister group to clade 3. Depending on the inference of the origin of the archeallum, either one or two T4P systems can be projected to the LACA. Under both of these scenarios, Clade 1 could be either a fast evolving outlier or a result of an HGT from an unknown bacterial source. Given the absence of methanococci, methanothermobacteria and thermococci, which comprise Clade 1, from Clade 2, accelerated evolution appears more likely.

### Genomic Neighborhoods of the Archaeal T4P

The genes linked to known components of T4P systems in archaeal genomes were identified in three iterations. Initially, we explored the neighborhoods of the archaeal genes that are annotated in arCOGs as components of archaeal T4P on the basis of previous studies (Pohlschroder et al., 2011; Jarrell and Albers, 2012; Lassak et al., 2012; Nair et al., 2013). All arCOGs found in the respective neighborhoods (three genes up- and downstream of the known T4P genes) in five or more archaeal genomes were included in the next iteration of the neighborhood analysis. After the third iteration, all neighborhoods were inspected manually, resulting in several arCOGs and individual proteins being added (in particular, when the respective genes were predicted to be secreted or membrane proteins, never occurred in a different gene context and were encoded in predicted operons with other arCOGs that could be confidently linked to T4P systems) and several arCOGs, mostly including predicted S-layer proteins, were excluded from the final set of T4P-associated arCOGs. The final list of the genomic loci encoding at least one of these genes is available in the supplementary material for all 168 analyzed archaeal genomes (Supplementary Table S4, neighborhoods).

As a result of this analysis, we identified 191 arCOGs linked to archaeal T4P (Supplementary Table S1, phyletic patterns). Altogether 5007 proteins could be confidently assigned to various T4P in archaea; of these proteins, 2817 (56%) are currently annotated as hypothetical or uncharacterized in public databases. Among these arCOGs, 5 represent VirB11 family ATPases, 7 FlaJ/TadC subfamilies, 90 archaellins/pilin subfamilies, 3 FlaK-like peptidase subfamilies and the rest are either known or putative components of these systems such as S-layer proteins, predicted minor pilins, predicted adhesins, and additional assembly proteins. Furthermore, several genes coding for secreted or membrane proteins and located in the neighborhoods of the respective T4P systems in some genomes but not currently included in the arCOGs were also included in to the list of the T4P-associated proteins. This list does not include several regulatory and signal transduction genes, which might be responsible for the regulation of T4P components expression (see below). Some of predicted S-layer proteins can be associated with T4P in some genomes but not in others, so they were not included in the final set but were separately listed as potential components of these system in particular genomes (Supplementary Table S5, potential components of T4P in the subset of genomes).

Most archaea possess several pilin genes that are located outside of the operons related to T4P, typically as singletons or tandem genes (Supplementary Table S4). At least two previous studies have shown that these are major pilin families (Ng et al., 2011; Esquivel et al., 2013). Accordingly, this feature was taken into account for prediction of major pilins families in the present work.

### Genes Associated with the T4P of Methanococci, Methanothermobacteria and Thermococci

**FIGURE 2:**
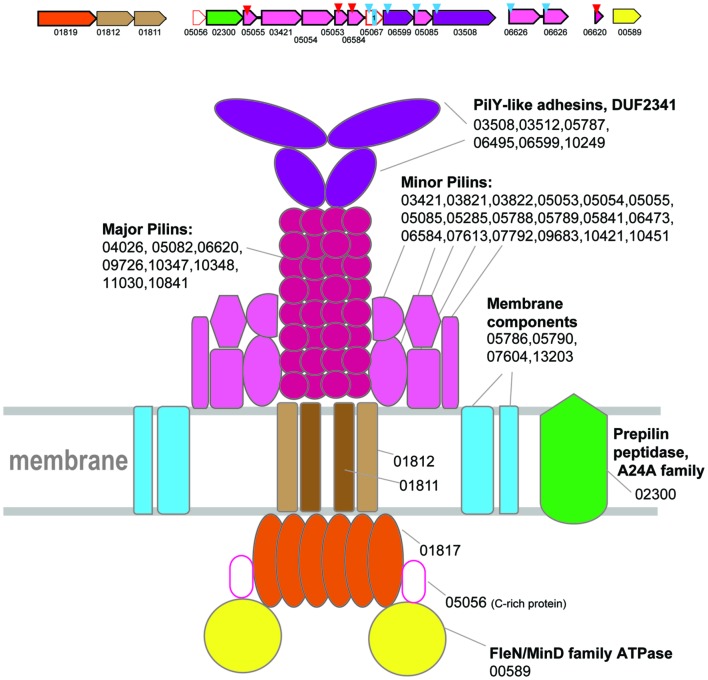
**Model of the structural organization of Clade 1 T4P system.** Hypothetical generalized model of clade 1 T4P system organization. A representative locus from Thermococcus is selected to show minor and major pilin by the colors corresponding to the model. The genes of a representative locus are shown by arrows. The scale of an arrow is roughly proportional to gene length. The arrow indicates direction of the respective gene. The red triangles above an arrow denote genes that encode FlaFind positive arCOG members. The blue triangles above an arrow denote genes that encode proteins predicted to be secreted. Small blue square with a number inside indicates a protein with corresponding number of TM domains. Color code for the genes is the following: secretion ATPases – orange, FleN/MinD family ATPase – bright yellow, TadC-like proteins – brown, major and minor pilin – magenta shades, adhesins – purple, prepilin signal peptidase – green; S-layer-like proteins are dark blue; Genes with no prediction of function but possible components of the system are white with red outline. For each component of this T4P system all arCOG families from all species corresponding to this component are listed.

ATPases from Clade 1 are present in class 1 methanogenes (methanococci, methanobacteria, and *Methanopyrus kandleri*) and thermococci (**Figure 1**, Supplementary Table S3). The only secretion ATPase identified in the genomes of Methanothermobacteria belongs to Clade 1 suggesting that the components of T4P identified in these genomes comprise a distinct, complete system, the only one present in Methanothermobacteria. The homologs of these genes and the genes co-localized with them in other genomes are therefore likely to belong to orthologous T4P systems in other archaea possessing an ATPase gene from this clade. This system has been studied experimentally in *M. maripaludis* S2 and shown to be involved in pili formation (Ng et al., 2011; Nair et al., 2013). In several species, the respective gene loci, which include several predicted pilins, have been described previously, together with many stand alone genes that contain class III signal peptides and are located outside of the main locus (Szabo et al., 2007). Based on this prediction, subsequent experimental analysis of this system in *M. maripaludis* S2 resulted in identification of the major pilin (MMP1685), a member of arCOG06620 family, and three minor pilins, MMP0233 (arCOG05055), MMP0236 (arCOG05053), and MMP0237 (arCOG06584), that are all essential for the pili formation (Ng et al., 2011). In Methanothermobacteria, only one gene from this system has been experimentally characterized, namely the major pilin MTH60 (arCOG10348) from Methanothermobacter thermautotrophicus (Thoma et al., 2008). In addition, there are two more genes of arCOG10348 family in the same genome and two more closely related paralogs from arCOG10347 (five genes altogether). Furthermore, a representative of arCOG06620 (MTH1102), the major pilin in Methanococci, is also encoded in this genome. Notwithstanding this diversity, there is only a limited number of families that are likely to correspond to major pilins associated with this system. Typically, these small (~70 aa) proteins are encoded by stand alone genes. Often, there are multiple paralogs in the same genome (Supplementary Tables S1 and S4). It seems likely that different sets of proteins from these families can be used as major pilins under different conditions; in the hypothetical model of the clade 1 T4P system organization, we include all these families (**Figure 2**).

Clearly, the T4P systems associated with this clade are highly complex and include multiple components, in addition to the ATPase, two TadC components and major pilins (Supplementary Figure S1; Supplementary Tables S1 and S4). Despite this complexity, it is possible to propose a model of this T4P system, based on the results of sequence analysis and by analogy with the thoroughly characterized archaellum (Jarrell and Albers, 2012; Albers and Jarrell, 2015; Banerjee et al., 2015) and multiple T4P and T2SS systems from bacteria (**Figure 2**). Specifically, in the respective loci we identified genes coding for two large proteins (arCOG03508 and others, see **Figure 2** and Supplementary Figure S1) that contain the DUF2341 domain. This domain is present in PilY adhesins associated with Type IV pili in *Pseudomonas aeruginosa* and is inferred to be located at the tip of the pilus (Nguyen et al., 2015). By analogy with *P. aeruginosa*, we hypothesize that these proteins perform a similar role in archaea (**Figure 2**). Other class III signal peptide containing proteins (often detected by FlaFind) that do not belong to major pilin families most likely are minor pilins. There are at least 5 such proteins encoded in the respective loci in most genomes. By analogy with minor archaellins in the archaellum, we place them close to the base of the pilus in the model although they also might form other decorations of the pilus such as hooks in the hami structure (Perras et al., 2015). Furthermore, at least one regulatory ATPase of the FleN/MinD family (arCOG00589), the dedicated prepilin peptidase EppA and at least two additional membrane proteins are often found in the respective loci (**Figure 2** and Supplementary Figure S1).

Overall, the T4P systems associated with this clade are on par with the complexity of several characterized bacterial T4P and type IV and II secretion systems (Burrows, 2012; Trokter et al., 2014; Berry and Pelicic, 2015), but are more complex than other archaeal T4P including even the archaellum.

### Genes Associated with the Archaellum

**FIGURE 3:**
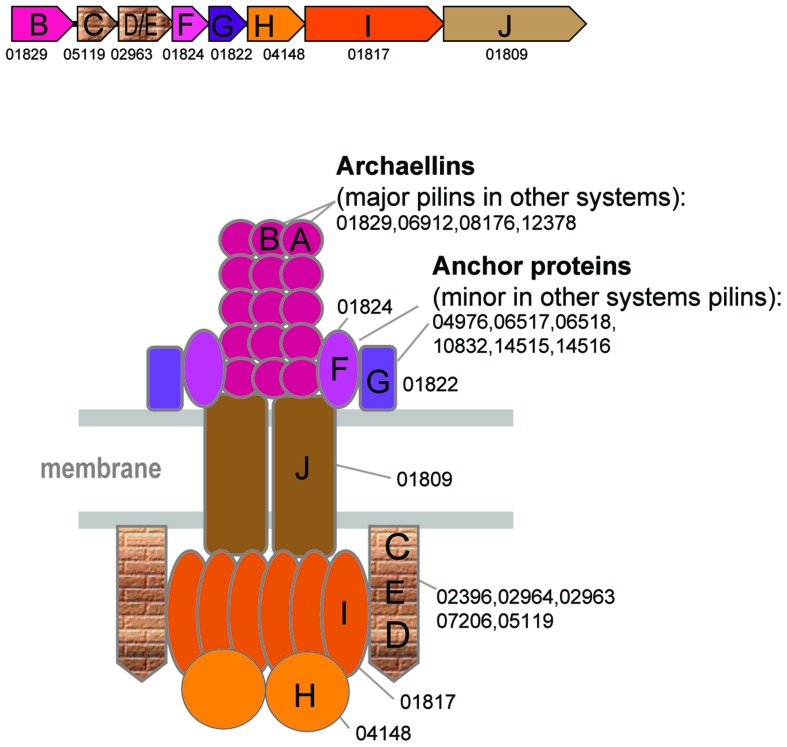
**Model of the structural organization of the archaellum.** Color scheme is the same as on the **Figure 2**. Additional color coding corresponds to the genes shown in the typical archaellum operon shown above the model.

The archaellum structure and associated genes are well studied and have been reviewed elsewhere (Chaban et al., 2007; Jarrell and Albers, 2012; Albers and Jarrell, 2015; Banerjee et al., 2015). However, in this work, a more extensive comparative analysis helped to identify additional archaellins and diverged FlaC/D/E components of the archaellum (**Figure 3** and Supplementary Figure S2). All archaea that have the archaellum (including *Nanoarchaeum* Nst1, the smallest genome where these genes are encoded) possess genes for its seven components, namely the ATPase (FlaI), an ATP-binding protein (FlaH), the membrane platform protein (FlaJ), minor archaellins FlaF, and FlaG (a single copy of each of the above, major archaellins of the FlaA and families (1–9 genes) and FlaC/E/D proteins (1–3 genes) (**Figure 3**, Supplementary Table S1). The FlaC/E/D proteins can be highly divergent in sequence and the respective genes are often fused giving rise to DE or CDE proteins (Supplementary Figure S2). Because of the lack of sequence similarity to euryarchaeal FlaC/E/D, the counterparts in Crenarchaeota and Thaumarachaea have been referred to as FlaX and an unknown gene, respectively. Using more sensitive methods for sequence comparison, we identified these components in all those species (Supplementary Tables S1, S4, and S6, HHpred). Archaellins, especially those of the FlaA/FlaB subfamily, are prone to duplications (e.g., up to nine paralogs in *Methanosphaerula palustris*) and sometimes are encoded in a different locus, often as stand alone genes (Supplementary Table S4).

In several Archaeoglobales species, the ancestral archaellum locus apparently was displaced by a horizontally transferred archaellum locus from different groups of archaea, in particular from a halobacterial source in *Archaeoglobus veneficus* SNP6 and from methanococci in *Ferroglobus placidus* (Supplementary Figure S2). In *Methanosarcina* there are two archaellum loci, which could be a product of a lineage specific duplication followed by functional specialization (Supplementary Figure S2).

In all archaellum-containing organisms, there is at least one additional T4P present (Supplementary Table S3).

### Genes Associated with T4P in Euryarchaea

The T4P systems associated with the VirB11 family ATPases of “euryarchaeal” Clade 2 (Supplementary Figure S3) are present in the majority of euryarchaeal groups at least in two copies suggestive of an ancestral duplication. More recent duplications have also occurred in several euryarchaeal lineages such as Archaeoglobales (Supplementary Figure S3). In many of these cases, only the locus containing ATPase/*tadC* genes, but not the one encoding pilins, is duplicated, suggesting that despite the apparent divergence following duplications both proteins can interact with the same pilin components. Paralogous ATPase/*tadC* operons could be differentially regulated. ATPases of this clade include also representatives of a few Thaumarchaea and Nanoarchaea (Supplementary Figure S3).

Furthermore, it has been shown that any of the six major pilins (PilA, all from arCOG02416) or their combination is sufficient for the biosynthesis of the pili that mediate surface adhesion in *Haloferax volcanii* (Esquivel et al., 2013). Several other pilins identified in this organism belong to arCOG02425 and arCOG02416, clear paralogs of those of arCOG02416 family and belong to the same supercluster in arCOGs (Supplementary Table S1). Subsequently, it has been demonstrated that expression of the PilB3 locus is required for assembly of PilA based pili and that it inhibits cell motility (Esquivel and Pohlschroder, 2014). This locus does not encode any of the potential pilins (Supplementary Figure S3). The orthologous locus in *Halobacterium salinarum* (OE2215R) is one of the two T4P systems shown to be expressed in this organism and have a distinct function in adhesion and thin filament formation (Losensky et al., 2014) (Supplementary Figure S3). This locus does not encode any predicted pilins either (Supplementary Table S4). These observations suggest that at least ATPase/TadC module in Halobacteria interacts with pilins encoded *in trans*. The link between the ATPase of this branch corresponding to PilB3 locus of *H. volcanii* with PilA-like pilins (of arCOG02416 and paralogs) also follows from the fact that in several archaeal genomes (e.g., *Methanocorpusculum labreanum*) all the respective genes are encoded in the same locus (Supplementary Figure S3).

**FIGURE 4:**
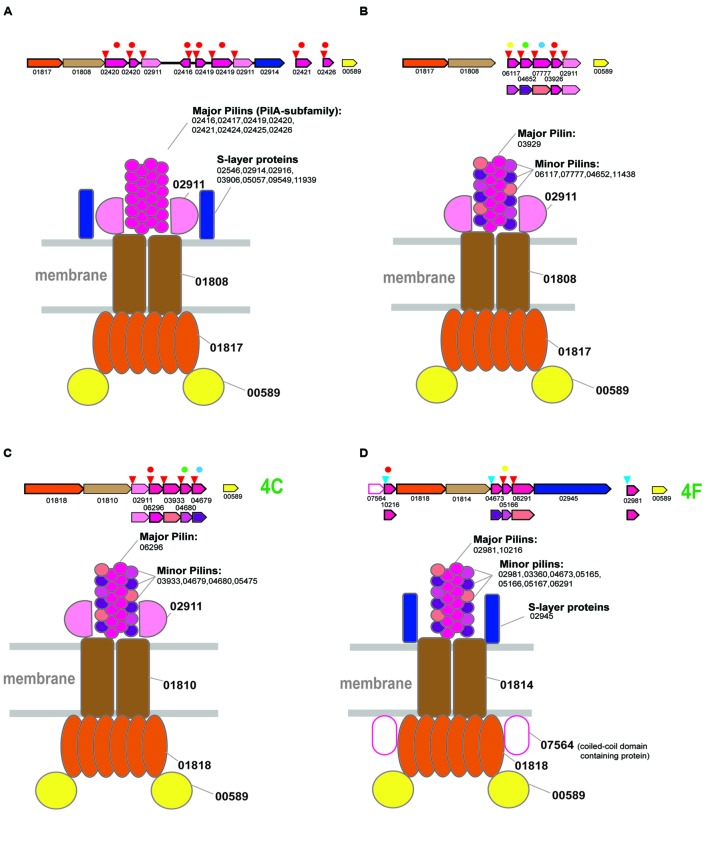
**Model of the structural organization of euryarchaeal T4P systems.** The color coding is the same as on the **Figure 2**. Additional color coding for pilins is shown underneath the respective operons. Homologous pilins in different systems are identified by the same colored circle above the respective genes. The red circles correspond to PilA family of predicted major pilins. **(A)** Model of T4P variant associated with branch 2. **(B)** Model of T4P variant associated with branch 2. **(C)** Model of T4P systems associated with subclade 4C. **(D)** Model of T4P systems associated with subclade 4F.

In agreement with the ancestral duplication hypothesis, there are two particular types of organization of T4P VirB11 family ATPases within this clade, the PilA-based pili (for example, PilB3 locus *H.volcanii* and respective PilA family major pilins **Figure 4A**) and another system typified by the PilB4 locus of *H volcanii* (**Figure 4B**). The ATPase/TadC module of the latter system is associated with its own set of pilins and is unlikely to be PilA-dependent given the presence of its own predicted major pilin (specifically, arCOG03926) which is also related, albeit more distantly, to the PilA family.

A single protein from arCOG02911 is shared between both systems, suggesting their structural similarity and common origin (Supplementary Figure S3). This gene is prone to duplications, often of variable length (~250–550 aa), and the encoded protein is usually identified by FlaFind, has a typical all beta stranded secondary structure suggestive of Ig-like fold, i.e., is a typical pilin. Given that the major pilins are already identified for both systems, we hypothesize that arCOG02911 is a minor pilin (**Figures 4A,B**).

The roles of the other pilins remain unclear. By analogy with the PilA-based system, we propose that they all could be structural components of the pilus filament (**Figure 4B**). Additional components might include an S-layer like protein, which is characteristic of PilA-based systems (**Figure 4A**, Supplementary Table S4).

Despite the presence of diverged paralogs of ATPases and cognate TadC proteins in many archaeal genomes, it appears that, at least under some conditions, they can share or exchange pilin sets. For example, in *Archaeoglobus veneficus*, closely related ATPases are associated with pilin sets linked to both system types, whereas in *Aciduliprofundum boonei*, two distinct ATPases are encoded in the same locus that contains only one *pilA*-like pilin gene (Supplementary Figure S3 and Supplementary Table S4). The ATPase of *H. volcanii* (HVO_1160) PilB4 locus has a distinct organization with additional N- and C- terminal domains (Supplementary Figure S4), which is specific for Halobacteria. Nevertheless, this ATPase is associated with same pilin set as many “standard” ATPase of this family in different genomes, again suggesting that the ATPase/TadC module evolves independently of the pilin set.

By analogy with clade 1, both systems associated with Clade 2 are expected to employ a FleN/MinD family ATPase (arCOG00589) which is usually encoded by a stand alone gene but is present in all these genomes. In bacteria, the FleN/MinD family ATPase is involved in the control of flagella assembly and localization (Dasgupta et al., 2000; Schuhmacher et al., 2015) and might have a function similar to that of FlaH in archaellum (Chaudhury et al., 2016).

Similarly to Clade 1 systems, many of euryarchaea from the Clade 2 have two *tadC* like genes in the respective loci, compatible with the hypothesis that Clade 1 is an extremely fast evolving derivative of the ancestral euryarchaeal system.

### Genes Associated with Distinct Groups from Subclade 4 T4P Clades 4C and 4F

The limited representation of the euryarchaea in subclades 4C and 4F suggests that these ATPases (most likely, with the cognate *tadC* gene) were horizontally transferred to the respective lineages from crenarchaea (**Figure 1**). However, several predicted pilins from these loci belong to the same families as the pilins associated with the typical euryarchaeal T4P from Clade 2 (Supplementary Figure S5), suggesting that these systems have a hybrid origin. Specifically, the ATPase/TadC module was most likely transferred from crenarchaea whereas the pilin sets evolved by duplication followed by diversification of the ancestral euryarchaeal pilins. This mode of pilin evolution is especially notable for the halobacteria-specific system from subclade 4C which, in addition to the three pilins shared with T4P (**Figure 4B**), also contains the signature pilin (arCOG02911) of all euryarchaeal T4P systems from the Clade 2. Because the only pilin with significant similarity to the PilA family belongs to arCOG06296, we hypothesize that it is the major pilin in this system. Because of all these features shared with clade 2 systems, especially the variant with several distinct pilin genes (**Figure 4B**), we propose the same structural layout for the system of subclade 4C (**Figure 4C**). The respective locus in *H. salinarum* also has been shown to be involved in adhesion and thin filament formation (Losensky et al., 2014).

The systems associated with subclade 4F are more diverse but also appear to be a hybrid, with ATPase and *tadC* that were likely transferred from crenarchaea and pilins of euryarchaeal origin (**Figure 4D** and Supplementary Figure S5). Secretion ATPases of this subclade often contain an additional, variable N-terminal cysteine-rich domain, which is especially prominent in methanomicrobia, where the cysteines could be involved in the formation of multiple disulfide bridges (Supplementary Figure S1). Similar to the euryarchaeal system depicted on the **Figure 4B**, this system appears to possess a dedicated major pilin (arCOG02981) of the PilA family. These proteins are most often encoded by stand alone genes but in a few cases were found in association with other components of this system (e.g., locus RCIX949-RCIX952 from *Methanocella arvoryzae*), which allowed us to link this family specifically to subclade 4F. Like many other major pilins, arCOG02981 genes are prone to tandem duplications (Supplementary Table S4). Another pilin of the PilA family (arCOG10216), found only in several halobacterial genomes, also could be a major pilin (**Figure 4D**). Another feature of this system that is shared with the one shown on **Figure 6A** is the frequent presence of a gene coding for a predicted S-layer like protein. Unique component of this halobacterial system (arCOG07564) is an intracellular protein with a coiled-coil domain. Presumably this is a regulatory subunit analogous to a coiled-coil protein from *Helicobacter pylori* essential for flagellum formation (Caly et al., 2010). The ATPase of this clade is also found in nanoarchaea, but the cognate pilins could not be identified in their genomes. Possibly as in case of euryarchaea described above these ATPases can be associated interact with other pilins present in nanoarchaeal genomes.

Overall, our data suggest that most of the euryarchaeal systems of Clade 2 and subclades 4C and 4F follow the same “grand” architectural plan (**Figure 4**), with minimal structural variations. These observations imply origin of the four major variants (**Figure 4**) from a simple ancestral four component system (consisting of secretion ATPase, TadC-like protein, minor pilin of arCOG02911 and major pilin of PilA family), mostly by duplication and subfunctionalization of the PilA family pilins.

### T4P Systems Specific to Sulfolobales and Desulfurococcales: Clades 4A, 4H, 4I, and 4J

**FIGURE 5:**
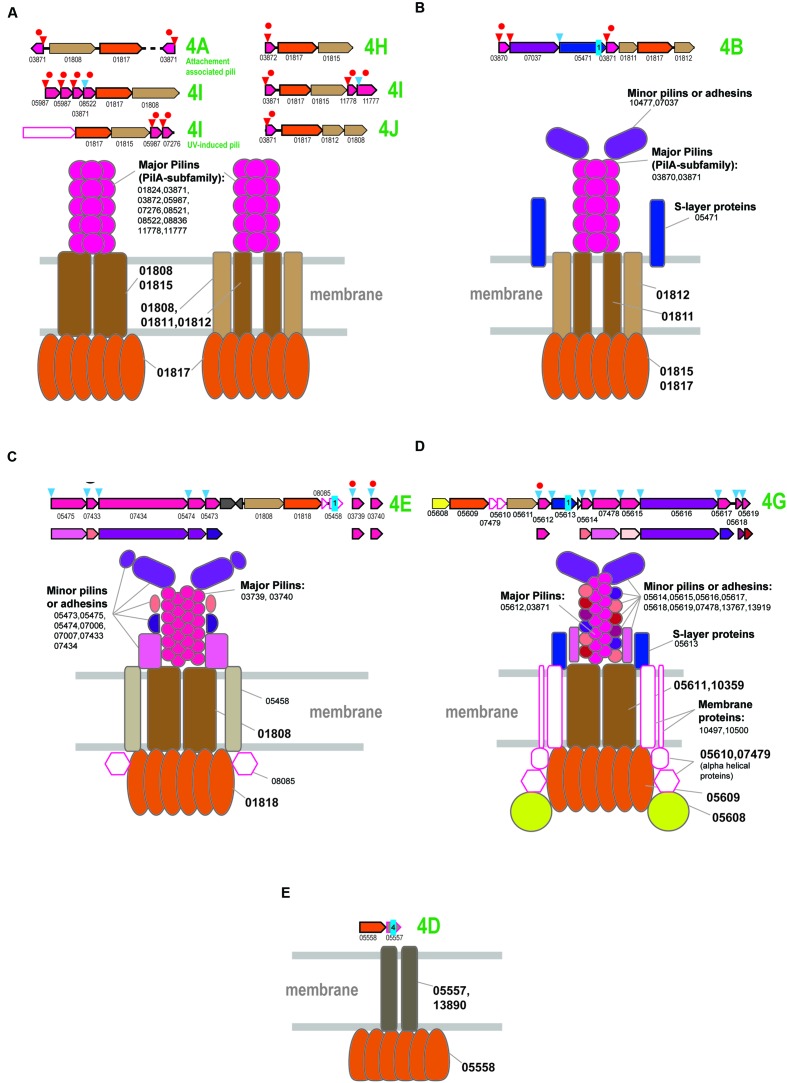
**Model of the structural organization of crenarchaeal T4P systems.** The systems that associated with different clades of subclade 4 are designated accordingly to the **Figure 1**. Designations and color coding is the same as on the **Figures 2** and **4. (A)** Model of T4P systems associated with clades 4A, 4H, 4I, and 4J, mostly present in Sulfolobales/Desulfurococcales. **(B)** Model of T4P systems associated with subclade 4B. **(C)** Model of T4P systems associated with subclade 4E. **(D)** Model of T4P systems associated with subclade 4G. **(E)** Model of T4P systems associated with subclade 4D.

Generally, the organization of the T4P loci associated with these subclades appears to be very diverse with a variable number of genes in the predicted operons. However, most of these proteins correspond to only three major components and thus the predicted model of the respective T4P systems appears to be very simple (**Figure 5A** and Supplementary Figure S6), with the TadC-like component encoded by either one or two gene and the vast majority of the pilins belonging to the PilA family. Some major pilins are duplicated within the locus, e.g., in the bindosome locus of Sulfolobus (subclade 4I) where all three pilins belong to the PilA family and some are encoded by stand-alone genes (**Figure 5A**, Supplementary Figure S6; Supplementary Table S4). Similar to the euryarchaeal T4P systems, the major pilins are likely to be compatible with most ATPases-TadC pairs. However, a more complex organization emerged in Desulfurococcales (subclade 4A), with 3 to 4 additional components the origin of which could not be traced. By analogy with the archaellum, we propose that these uncharacterized proteins comprise a minor pilin set (**Figure 5**). In Thaumarchaea (clades 4A and 4I), additional components are also present and correspond to a potential S-layer protein or adhesin.

The subclades 4A and 4I also include several small groups of ATPases from Thermoproteales. The respective T4P loci usually have a simple organization, with the exception of *Thermoproteus uzoniensis* which contains at least 6 additional unique components, mostly proteins that could function as minor or major pilins.

Two characterized T4P systems of Sulfolobus, the attachment-associated pili system (subclade 4A) and UV-induced pili (subclade 4I) are predicted to have a rather simple organization (**Figure 5A** and Supplementary Figure S6). In contrast, the third T4P system in these organisms, the bindosome (subclade 4I), appears to include several additional components, among which there are two membrane proteins (arCOG07264 and arCOG07329) and a specific ~500 aa secreted protein (arCOG08524) that could be either an adhesin or a S-layer protein. Both membrane proteins are present in several other archaea, where they are not encoded in the T4P loci and thus might not be essential for the bindosome assembly.

In summary, it appears that, despite the diversity of the gene content of the T4P loci in Sulfolobales and Desulfurococcales, many genes could perform regulatory functions and/or could be dispensable. All the predicted pilins associated with these systems belong to the PilA family and are likely to be major pilins that can assemble into the pilus filament in any combinations. In most cases, the organization of the system can be approximated by three components only (**Figure 5A**) and often the major pilins are encoded in the predicted operon with the ATPase, TadC or both (Supplementary Figure S6). However, it appears likely that these major pilins can interact with diverse ATPase/TadC modules *in trans*, thus increasing the combinatorial diversity of the T4P systems.

### T4P Systems Specific for Thermoproteales: Clades 4B, 4D, 4E, and 4G

In Thermoproteales, the T4P systems are extremely diverse and some seem to have experienced a period of a very fast evolution (**Figure 1** and Supplementary Figure S7). The 4B clade ATPases correspond to T4P systems with a rather straightforward organization (**Figure 5B**). In addition to the ATPase, they have two genes for TadC-like components and two genes encoding PilA family pilins. Both are expected to be major pilins, and each is probably sufficient to form a functional pilus. Additionally, some of these loci encode a potential S-layer protein (arCOG05471), which is predicted to be attached to the membrane via a single C-terminal transmembrane domain and contains several conserved cysteines potentially involved in disulfide bond formation. Orthologs of this protein are present also in other T4P loci. Another additional component is a large signal peptide containing protein that could be either a minor pilin or an adhesin (**Figure 5B**).

Systems associated with subclade 4E appear to be the most conserved ones as indicated by the short branches on the ATPase tree (**Figure 1**) and highly similar sets of components in distantly related genomes (**Figure 5C** and Supplementary Figure S7). Major pilins associated with the subclade 4E systems are likely members of arCOG03739 and arCOG03740 that, on a few occasions, are encoded in the same locus (e.g., GI: 374327804 in Pyrobaculum 1860) but most often are found as stand-alone genes (Supplementary Table S4). These pilins belong to the PilA family (**Figure 5C**, Supplementary Figure S7; Supplementary Tables S1, S4, and S6). Other secreted components could be either minor pilins or adhesins (**Figure 5C** and Supplementary Figure S7). In addition, there is a membrane protein and another cytoplasmic component encoded in these loci (**Figure 5C** and Supplementary Figure S7). Homologs of the respective arCOGs (05475 and 05473) are also present in a locus with an uncertain affiliation of the ATPase that is found in the genomes of Vulcanisaeta and Caldivirga species (Supplementary Figure S7; Supplementary Tables S1 and S4). The ATPase and TadC components are not encoded in these loci; the best candidate ATPase in Vulcanisaeta and Caldivirga to interact with these pilins is the one from the subclade 4A which is encoded next to a cytoplasmic and membrane protein similarly with other loci associated with the subclade 4E. Loci with similar gene compositions are also present in two Thermofilum species. These loci can be tentatively linked to the only ATPase clade specific for two Thermofilum species that is not associated with other pilins (**Figure 1** and Supplementary Figure S7). The predicted minor pilin of arCOG05475 can be considered a signature for this system.

The systems associated with subclade 4G appear to be highly complex, especially in the Pyrobaculum-Thermoproteus group. The latter have a distinct diverged ATPase of arCOG05609 and a diverged TadC family protein of arCOG05611 (Supplementary Figure S7). The only predicted pilins (arCOG05612) that show detectable sequence similarity with pilins from other systems belong to the PilA family and apparently are the major pilins associated with this system. Most of the predicted pilins show no similarity to the corresponding pilins associated with ATPases from Vulcanisaeta and Caldivirga which also belong to the 4G clade. The only exception are the clearly identifiable pilins of the PilA family assigned to arCOG03871 (Supplementary Figure S7). These observations emphasize the extremely fast evolution of the system components. Among the multiple components of this system in Pyrobaculum-Thermoproteus, there are two large proteins. One of these, containing a C-terminal transmembrane domain, is probably an S-layer-like protein whereas the other is a signal peptide containing protein, possibly an adhesin or a minor pilin. The diverse, smaller proteins are likely to be minor pilins (**Figure 5C**). Almost all components of these systems contain multiple, conserved cysteines that are expected to form disulfide bridge and thus additionally stabilize the proteins. The loci in Vulcanisaeta and Caldivirga additionally encode two membrane proteins (arCOGs 10497 and 10500) (Supplementary Figure S7). These two proteins are also found in several Pyrobaculum species but are encoded separately (Supplementary Table S4). Nevertheless, these proteins are likely parts of the assembled T4P systems (**Figure 5C**). All these systems encode a distinct putative regulatory FleN/MinD family ATPase (arCOG05608). Two additional components that are specific for Pyrobaculum/Thermoproteus species are small, alpha-helical intracellular proteins whose function is unclear (**Figure 5C**).

Finally, the systems associated with ATPases from the subclade 4D are the most unusual ones. They contain only two components and no potential pilins or any genes encoding secreted proteins are ever found in the respective loci (**Figure 5D** and Supplementary Figure S7). The second component of the system is a 4 or 5 TM domain-containing proteins can belong either to arCOG05557 or arCOG13890 which show no significant similarity to each other or any other membrane proteins. These proteins might be highly diverged derivatives of TadC, in particular the short form (e.g., arCOG01812). In Pyrobaculum and Thermoproteus species, the respective ATPase (arCOG05558) lacks the C-terminal domain but contains an additional, small N-terminal domain with several conserved cysteines that could be involved in the formation of disulfide bonds (Supplementary Figure S1). The function of this system remains unknown. The possibility remains that these systems employ still unidentified pilins encoded in different loci or any subset of major and minor pilins from other systems; alternatively, these systems might have changed their function dramatically and do not function as T4P. The latter possibility is especially attractive considering presence of a gene for a TFIIB (transcriptional factor IIB) homolog (arCOG05559) in the same predicted operon (Supplementary Figure S7). This TFIIB variant is specific for the Pyrobaculum-Thermoproteus group and, typical of TFIIB, contain a Zn finger and cyclin domains. Furthermore, in Thermoproteus, the ATPase, membrane protein and TFIIB are part of a predicted operon which also encodes the RPB8 subunit of the RNA polymerase; in Pyrobaculum, the RPB8 gene has an orientation opposite to that of the former three genes, so that they all could be co-transcribed as well (Supplementary Table S4). These observations suggest that this system could be functionally distinct form other T4P and might be involved in some transcription-related pathway.

Altogether, it appears that the T4P-like systems in Thermoproteales have experienced bursts of diversification at different points of evolution of this lineage, both with respect to the fast evolution of most of the components and the emergence of complex architectures with several distinct pilin sets and acquisition of additional components, such as membrane proteins. This diversity is in a sharp contrast to the multiple but simpler organized T4P systems of Sulfolobales and Desulfurococcales.

### Prepilin Peptidases and Associated Components

Class III signal peptides are cleaved by a dedicated type IV signal peptidase of the A24 family which upon processing removes only the positively charged N-terminus of the signal peptide, leaving a hydrophobic domain of about 20 amino acids attached at the N-terminus of the mature protein (Albers and Driessen, 2002; Albers et al., 2003; Craig et al., 2004), which is important for filament formation of type IV pilins. At least one peptidase of this family is present in the majority of the archaeal genomes that encode a secretion VirB11 family ATPase, with the exception of two nanoarchaea and Candidatus *Nitrosopumilus* AR2 (Supplementary Table S1). There are two subfamilies of these peptidases, namely the 5 TM domain form (arCOG02298 and arCOG07367) and the 9 TM domain form (arCOG02300). The most abundant one, known as PibD or FlaK, is the 5 TM form. Whereas PibD has been shown to have a broad substrate range (pilins, archaellins, substrate-binding protein (Sulfolobales and *Hfx. volcanii*), FlaK in *M. maripaludis* specifically processes only the archaellins (Albers et al., 2003; Szabo et al., 2007; Tripepi et al., 2010; Hu et al., 2011). The 9 TM variant, EppA, is present only in the organisms associated with clade 1 and are often found in the respective gene context (Supplementary Figure S7). It has been shown that EppA, but not FlaK, is responsible for the signal peptide processing of pilins in *M. maripaludis* (Szabo et al., 2007).

In addition, we noticed a strong link between surface peptidases of the transglutaminase family, namely arCOG02164 and arCOG09555, with clade 1 T4P systems in Methanococci and Methanothermobacteriales, respectively, and arCOG03450 with euryarchaeal T4P in Methanomicrobiales (Supplementary Table S4). The role(s) of these peptidases are unknown; they might be involved in further processing or degradation of pilins.

### Regulation of T4P Expression

Little is known about the specific regulation of T4P assembly and disassembly processes. The regulatory systems so far have been studied only with respect of the archaellum regulation in Sulfolobus. Comparative genomic analysis allows one to examine the distribution of this particular regulatory system in other archaeal genomes, in order to assess the strength of its affinity with T4P and/or archaellum loci and predict other potential regulatory components.

The most abundant regulators associated with T4P systems are ATPases, of the FlaH (arCOG04148) and the more diverse FleN/MinD-like (arCOG00589) families (Supplementary Tables S1 and S4). The first one is a dedicated archaellum component which directly interacts with FlaI and is essential for the motor assembly (Chaudhury et al., 2016). It is a stable component of the respective operons (Supplementary Figure S2) whereas the second one is a more general regulator with multiple paralogs (Supplementary Table S1) that apparently can regulate a variety of T4P systems. In some case, the FleN/MinD-like ATPases are encoded in the same predicted operon T4P systems but more often they are encoded separately or in a predicted operon with several unrelated intracellular or membrane proteins (Supplementary Tables S1 and S4).

The FlaH-like ATPases show extensive sequence similarity to the much larger KaiC family ATPases many of which (e.g., arCOG01171, arCOG01174, arCOG01175) also show a strong association with a variety of T4P systems. These ATPases are encoded in the vicinity and often in the predicted operons of the archaellum, clade 2, and subclade 4B T4P systems (Supplementary Table S4). A few other families of KaiC ATPases (arCOG01172, arCOG01173) also could be co-transcribed with the VirB11/TadC operon in Methanomicrobia (**Figure 5**, Supplementary Table S4). The ATPases of this family have been best studied in cyanobacteria in the context of the circadian clock (Egli and Johnson, 2013; Axmann et al., 2014), but are also known to regulate the expression of gas vesicles in halobacteria (Pfeifer et al., 2001). These ATPases typically possess auto-phosphorylation and de-phosphorylation activity and depending on the phosphorylation state interact with a second component of the system, which sends a signal further down the regulatory pathway (Egli and Johnson, 2013; Axmann et al., 2014). In most cases, there is no evidence of a second component with which KaiC-like ATPase could interact but in several genomes this component could be predicted. One such case is observed for the T4P system associated with subclade 4B which contains the pair of genes for KaiC-like ATPase of arCOG01174 family and an alpha helical protein (arCOG03758) distantly similar to eukaryotic DEATH domain (KSM and EVK unpublished) (Supplementary Figure S7). Given that in many other archaeal genomes the arCOG01174 genes are associated with KaiB-like component of thioredoxin family, the DEATH domain protein might functionally substitute the KaiB-like protein and could be involved in an archaeal oscillation system, which regulates the T4P expression.

Another characterized regulatory system includes repressors of archaella expression genes, the ArnA protein containing a FHA domain (arCOG05332) and the ArnB protein containing a vWA domain (arCOG02900) (Reimann et al., 2012). The presence of a FHA domain suggests that this system is part of a general signal transduction mechanism based on phosphorylation by a S/T protein kinase and dephosphorylation by an associated phosphatase (Mahajan et al., 2008; Alber, 2009). Indeed, it has been shown that both proteins are phosphorylated and strongly interact *in vivo* (Reimann et al., 2012). The ArnA and ArnB proteins are often encoded together in a predicted operon but are never present in T4P loci or archaellum (Supplementary Table S4). The vWA domain-containing proteins of the ArnB subfamily are more often associated with MoxR/GvpN ATPases suggesting that there is an alternative regulatory pathway that involves the same vWA domain. The pair of genes coding for vWA domain containing protein (arCOG02900) and GvpN-like ATPase (arCOG00441) is strongly linked to thaumarchaeal T4S loci of subclade 4A (Supplementary Figure S6 and Supplementary Table S4). In bacteria, vWA domain-containing proteins, such as TadG, have been also identified as components of pili where they are hypothesized to anchor the pilus to the membrane (Wang and Chen, 2005; Tomich et al., 2007).

Another regulatory system consisting of the ArnR and ArnR1 (both from arCOG05969, pfam13463 family) is involved in the regulation of *flaB*, the filament protein of the archaellum, and accordingly, deletion mutants of these regulatory genes are immotile (Lassak et al., 2013). The *arnR*-like genes are often present in the archaellum loci but only in Sulfolobales and Desulfurococcales (Supplementary Table S4). The proteins of this family are apparently associated with the membrane because they contain an N-terminal TM domain. Another experimentally characterized transcriptional regulator belongs to the Lrs14 family (arCOG02242, pfam01978 family) (Orell et al., 2013). Most archaeal genomes encompass several paralogs of this family (Supplementary Table S1). In *Sulfolobus acidocaldarius*, at least three of these regulators are involved in T4P regulation. One of these, Saci0446 (AbfR1), controls expression of both the archaellum and attachment-associated pili, whereas two others, Saci1223 and Saci1242, are important for biofilm formation (Orell et al., 2013). These regulators are never encoded in the T4P or archaellum loci (Supplementary Table S4).

Many diverse (predicted) transcriptional regulators that have not been studied experimentally were identified in the T4P or archaellum loci in the course of the present analysis (Supplementary Table S1 and S4). The most common among them is arCOG01981, transcription initiation factor TFIIB homolog, which is found in many T4P loci (Supplementary Table S4). This protein contains a Zn finger and two cyclin domains (in contrast to the single cyclin domain in arCOG05559 that is specifically implicated in the function of subclade 4D systems as indicated above). The next most frequent regulator is arCOG00381 (pfam11748 family), a membrane-bound transcriptional regulator, which is likely to be involved in the regulation of the predicted major pilins of arCOG02425 in Halobacterial T4P of clade 2. The arCOG03422 transcriptional regulator (pfam07381) is often found in archaellum loci of Methanococci, Thermococci and Ferroglobus (Supplementary Figure S2). The arCOG03422 genes almost always are associated with arCOG05058 genes which encode a SAM-dependent methyltransferase fused to an N-terminal dimerization domain (Supplementary Table S4) suggesting a strong functional link between the two proteins. Several other families of transcriptional regulators show affinity with specific components of T4P systems. For example, arCOG01057 (pfam01638) is linked to FleN/MinD-like ATPase of arCOG00589 (Supplementary Table S4), whereas arCOG03067 (pfam12840) is often found close to *flaB* genes in many euryarchaea and thus might be functionally analogous to ArnR regulators in crenarchaea.

The presence of chemotaxis genes, which are found only in euryarchaea, in the vicinity of archaellum has been noticed and studied previously (Schlesner et al., 2009). In particular, it has been shown that CheF proteins (arCOG02394) provide an interface between FlaDCE proteins and several proteins of the bacteria-like chemotaxis apparatus (Schlesner et al., 2009). The CheF genes are often located within archaellum loci in the majority of Halobacteria, Methanomicrobia and in some Archaeoglobi (Supplementary Table S4). There is no evidence that chemotaxis system are involved in the regulation of other T4P systems.

### Cytoplasmic Proteins Associated with T4P Systems

Numerous genes encoding functionally uncharacterized cytoplasmic proteins show strong links to T4P loci. These proteins might be auxiliary structural or regulatory components or could be co-expressed with T4P components as parts of a larger regulon that, in addition to T4P, might include other cellular systems or metabolic pathways. Several of these are predicted metal-binding proteins, including cysteine rich arCOG05056 family associated with clade 1 T4P systems of Methanococci, membrane proteins of arCOG07264 containing an N-terminal Zn ribbon associated with Sulfolobus bindosome loci, and arCOG06883 proteins with a double Zn-ribbon domain mostly present in the T4P loci of Thaumarchaea. Alpha helical proteins of arCOGs 05610 and 7479 are specifically associated with subclade 4G, a small alpha+beta protein of arCOG08085 is linked to clade 4E, and a coiled-coil arCOG07564 protein is found in subclade 4F loci (Supplementary Figures S5 and S7).

Conversely, some proteins from well characterized families are encoded in the T4P loci and might be parts of a complex regulon together with the respective T4P systems. In particular, an FtsZ-family GTPase (arCOG02202) and a squalene cyclase (arCOG03396) are encoded in the clade 1 loci in most Thermococci as previously described (Szabo et al., 2007). We also observe the same family *ftsZ*-like gene in the context of subclade 4F T4P systems in Halobacteria (Supplementary Table S4). Also in clade 1 loci in methanobacteriales and methanococci contain a gene for a PP-loop ATPase of the diphthamide synthase family (arCOG00035) that is likely co-transcribed with the T4P components (Supplementary Table S4). Finally, roadblock/LC7 domain containing proteins of arCOG02603 are often encoded in a variety of T4P loci (Supplementary Table S4).

## Discussion

Phylogenomic analysis of archaeal T4P described here reveals remarkable abundance and diversity of these systems. Multiple cases of horizontal transfer of T4P loci between archaea were detected but at least one T4P system can be inferred to have been present in the last common ancestor of the extant archaea. Perhaps the most striking observation is that these systems that are generally responsible for the interaction of microbial cells with various surfaces and with each other are especially abundant in hyperthermophiles. Moreover, T4P in the hyperthermophilic order *Thermoproteales* reach extreme diversity far exceeding that in other archaea. Additional bursts of rapid evolution of T4P appear to have occurred in individual lineages such as those of *Thermoproteus uzoniensis* or *Methanomethylovorans hollandica*. Archaeal hyperthermophiles typically live in turbulent, often boiling waters and are not thought to aggregate or to adhere to surfaces. The presence of numerous, extremely diverse T4P in these organisms challenges this common knowledge and suggests that major aspects of the hyperthemophile biology remain unknown.

Altogether, present analysis confidently links ~5000 archaeal proteins to T4P systems; more than half of these proteins (56%) are currently annotated as hypothetical in public databases. This list is conservative and does not include many genes that might be lineage-specific components of T4S systems. Based on comprehensive comparative analysis of sequences of the protein components and genomic neighborhoods of archaeal T4P, we propose detailed models of structural organization of the 10 most abundant T4P systems. In addition to the discrimination between major and minor pilins, these models include system-specific ancillary components such as S-layer proteins, adhesins, and various membrane and intracellular proteins. In most of the systems, dedicated major pilin families are identified, including numerous stand alone major pilin genes of PilA family. Evidence is presented that secretion ATPase and the cognate TadC components of T4P can work with different pilin sets resulting in modular evolution and extensive combinatorial diversity. We also predict many regulators of expression and activity of T4P including KaiC family ATPases, vWA domain containing proteins and respective MoxR/GvpN ATPases, TFIIB homologs and multiple, unrelated transcription regulators, some of which are associated with specific T4P systems.

The results of comparative analysis presented here are expected to facilitate experimental characterization of the T4P systems which might uncover major new aspects of archaeal biology.

## Author Contributions

Data analysis: KM. Contributed to the writing of the manuscript: KM, EK, and S-VA.

## Conflict of Interest Statement

The authors declare that the research was conducted in the absence of any commercial or financial relationships that could be construed as a potential conflict of interest.

## Footnotes

1 ftp://ftp.ncbi.nlm.nih.gov/genomes/refseq/

2 Availability: arCOGs database files are available at: ftp://ftp.ncbi.nlm.nih.gov/pub/wolf/COGs/arCOG/; all Figures and supplementary files can be found in the original formats (PDF, XLSX and DOCX) at: ftp://ftp.ncbi.nlm.nih.gov/pub/wolf/_suppl/T4P/
