# Supplementary material for: Diversity and Evolution of Type IV pili Systems in Archaea
Source: Front Microbiol. 2016 May 6;7:667. doi: 10.3389/fmicb.2016.00667 (PMC4858521; doi:10.3389/fmicb.2016.00667)
Supplement: Supplementary file 1 [file Presentation_1.ZIP › Supplementary File 1.docx]

Supplementary File 1. Complete tree for archaeal secretion ATPases in Newick format

(((327310829_Thermoproteus_uzoniensis_768_20_uid65089:0.69649,((352682104_Thermoproteus_tenax_Kra_1_uid74443:0.00768,327310486_Thermoproteus_uzoniensis_768_20_uid65089:0.01704)0.958:0.09863,(((126460754_Pyrobaculum_calidifontis_JCM_11548_uid58787:0.02912,145590298_Pyrobaculum_arsenaticum_DSM_13514_uid58409:0.03159)0.940:0.02113,18312170_Pyrobaculum_aerophilum_IM2_uid57727:0.03011)0.732:0.01713,119872579_Pyrobaculum_islandicum_DSM_4184_uid58635:0.02170)0.925:0.09779)1.000:0.70595)0.962:0.21792,((((118575861_Cenarchaeum_symbiosum_A_uid61411:0.77651,(407465042_Candidatus_Nitrosopumilus_AR2_uid176130:0.37313,408404599_Candidatus_Nitrososphaera_gargensis_Ga9_2_uid176707:0.35364)0.723:0.10239)0.249:0.08158,((((227831595_Sulfolobus_islandicus_L_S_2_15_uid58871:0.02026,15899403_Sulfolobus_solfataricus_P2_uid57721:0.03545)1.000:0.47836,((284998518_Sulfolobus_islandicus_L_D_8_5_uid43679:0.05439,15897080_Sulfolobus_solfataricus_P2_uid57721:0.02587)1.000:0.33480,((146304852_Metallosphaera_sedula_DSM_5348_uid58717:0.12510,330834048_Metallosphaera_cuprina_Ar_4_uid66329:0.11177)0.998:0.20366,(70607238_Sulfolobus_acidocaldarius_DSM_639_uid58379:0.23612,15921686_Sulfolobus_tokodaii_7_uid57807:0.17832)0.982:0.14697)0.138:0.08147)0.997:0.25211)0.890:0.08674,305662566_Ignisphaera_aggregans_DSM_17230_uid51875:0.86747)0.537:0.04706,(325969268_Vulcanisaeta_moutnovskia_768_28_uid63631:0.09549,307595032_Vulcanisaeta_distributa_DSM_14429_uid52827:0.06070)1.000:0.64562)0.881:0.11543)0.967:0.13723,(347522903_Pyrolobus_fumarii_1A_uid73415:0.56797,((124027831_Hyperthermus_butylicus_DSM_5456_uid57755:0.37932,347523837_Pyrolobus_fumarii_1A_uid73415:0.24234)0.922:0.11816,(((549455157_Aeropyrum_camini_SY1___JCM_12091_uid222311:0.02593,118431156_Aeropyrum_pernix_K1_uid57757:0.04494)0.991:0.15571,(429217556_Caldisphaera_lagunensis_DSM_15908_uid183486:0.24117,302348017_Acidilobus_saccharovorans_345_15_uid51395:0.21530)0.998:0.22340)1.000:0.27257,((297527441_Staphylothermus_hellenicus_DSM_12710_uid45893:0.02133,126465881_Staphylothermus_marinus_F1_uid58719:0.02492)0.998:0.25893,(389860619_Thermogladius_1633_uid167488:0.30857,296242533_Thermosphaera_aggregans_DSM_11486_uid48993:0.36663)0.649:0.10899)0.995:0.23004)0.803:0.09357)0.964:0.11593)0.819:0.08048)0.157:0.05345,(557694748_Candidatus_Caldiarchaeum_subterraneum_uid227223:0.75204,146303966_Metallosphaera_sedula_DSM_5348_uid58717:0.86945)0.200:0.06955)0.130:0.02077)0.820:0.02438,(((((159041769_Caldivirga_maquilingensis_IC_167_uid58711:0.14112,(325969315_Vulcanisaeta_moutnovskia_768_28_uid63631:0.03756,307595080_Vulcanisaeta_distributa_DSM_14429_uid52827:0.00015)0.991:0.09870)1.000:0.23895,((227829233_Sulfolobus_islandicus_L_S_2_15_uid58871:0.13222,(70608025_Sulfolobus_acidocaldarius_DSM_639_uid58379:0.21966,(15920739_Sulfolobus_tokodaii_7_uid57807:0.12470,(332797853_Acidianus_hospitalis_W1_uid66875:0.10851,(146303433_Metallosphaera_sedula_DSM_5348_uid58717:0.04238,330835387_Metallosphaera_cuprina_Ar_4_uid66329:0.06290)0.977:0.06096)0.933:0.03914)0.780:0.02332)0.923:0.07492)1.000:0.33558,(408405527_Candidatus_Nitrososphaera_gargensis_Ga9_2_uid176707:0.16461,(407462477_Candidatus_Nitrosopumilus_koreensis_AR1_uid176129:0.12682,(407464826_Candidatus_Nitrosopumilus_AR2_uid176130:0.05237,340344875_Nitrosoarchaeum_koreensis_MY1_MY1:0.08528)0.934:0.07212)1.000:0.21796)0.997:0.18339)0.892:0.07857)0.660:0.03410,(((530780318_Thermofilum_1910b_uid215374:0.07071,119719356_Thermofilum_pendens_Hrk_5_uid58563:0.00015)0.998:0.18813,((296242114_Thermosphaera_aggregans_DSM_11486_uid48993:0.05360,(320100495_Desulfurococcus_mucosus_DSM_2162_uid62227:0.01762,(390937986_Desulfurococcus_fermentans_DSM_16532_uid75119:0.00381,218883477_Desulfurococcus_kamchatkensis_1221n_uid59133:0.00014)0.930:0.02177)0.987:0.06106)0.986:0.07967,(389861548_Thermogladius_1633_uid167488:0.07473,(297526959_Staphylothermus_hellenicus_DSM_12710_uid45893:0.00375,126466359_Staphylothermus_marinus_F1_uid58719:0.00383)0.997:0.10228)0.881:0.03739)0.998:0.15998)0.992:0.14429,((549456029_Aeropyrum_camini_SY1___JCM_12091_uid222311:0.03658,118431780_Aeropyrum_pernix_K1_uid57757:0.01144)1.000:0.29201,(385805740_Fervidicoccus_fontis_Kam940_uid162201:0.27219,(124027967_Hyperthermus_butylicus_DSM_5456_uid57755:0.20546,(305664069_Ignisphaera_aggregans_DSM_17230_uid51875:0.19871,347523695_Pyrolobus_fumarii_1A_uid73415:0.14264)0.877:0.04201)0.601:0.04239)0.803:0.06125)0.484:0.03725)0.876:0.05988)0.971:0.07403,(((347524152_Pyrolobus_fumarii_1A_uid73415:0.32337,(((126459954_Pyrobaculum_calidifontis_JCM_11548_uid58787:0.09514,119872898_Pyrobaculum_islandicum_DSM_4184_uid58635:0.02936)0.841:0.03812,(327310805_Thermoproteus_uzoniensis_768_20_uid65089:0.14699,352682328_Thermoproteus_tenax_Kra_1_uid74443:0.05634)0.863:0.03253)1.000:0.31503,(159041007_Caldivirga_maquilingensis_IC_167_uid58711:0.41425,(307595209_Vulcanisaeta_distributa_DSM_14429_uid52827:0.06034,325969464_Vulcanisaeta_moutnovskia_768_28_uid63631:0.06070)0.997:0.18462)0.919:0.10418)0.995:0.16157)0.908:0.10555,(156937799_Ignicoccus_hospitalis_KIN4_I_uid58365:0.40071,(305664052_Ignisphaera_aggregans_DSM_17230_uid51875:0.28172,(302348099_Acidilobus_saccharovorans_345_15_uid51395:0.24424,124027110_Hyperthermus_butylicus_DSM_5456_uid57755:0.19604)0.822:0.10647)0.996:0.22105)0.873:0.08997)0.651:0.04113,(530780661_Thermofilum_1910b_uid215374:0.10545,119719717_Thermofilum_pendens_Hrk_5_uid58563:0.08461)1.000:0.25888)0.945:0.07090)0.868:0.05007,(((297619915_Methanococcus_voltae_A3_uid49529:0.12882,(((336121696_Methanothermococcus_okinawensis_IH1_uid51535:0.08606,150401577_Methanococcus_aeolicus_Nankai_3_uid58823:0.12230)0.698:0.01336,(45357603_Methanococcus_maripaludis_S2_uid58035:0.03660,150399785_Methanococcus_vannielii_SB_uid58767:0.05038)0.996:0.06238)0.381:0.01760,((296109003_Methanocaldococcus_infernus_ME_uid48803:0.05412,(256810973_Methanocaldococcus_fervens_AG86_uid59347:0.02589,(289192866_Methanocaldococcus_FS406_22_uid42499:0.01518,15668962_Methanocaldococcus_jannaschii_DSM_2661_uid57713:0.00014)0.659:0.01316)0.842:0.01532)1.000:0.11719,333911005_Methanotorris_igneus_Kol_5_uid67321:0.03693)0.861:0.03044)0.850:0.03539)1.000:0.31771,(((148642657_Methanobrevibacter_smithii_ATCC_35061_uid58827:0.11805,(509154390_Methanobrevibacter_AbM4_uid206516:0.20484,288559779_Methanobrevibacter_ruminantium_M1_uid45857:0.15414)0.916:0.06241)0.960:0.07249,((312136704_Methanothermus_fervidus_DSM_2088_uid60167:0.09575,(15679696_Methanothermobacter_thermautotrophicus_Delta_H_uid57877:0.02347,(333988139_Methanobacterium_SWAN_1_uid67359:0.04019,566003565_Methanobacterium_MB1_uid231690:0.05558)0.867:0.02913)0.973:0.07425)0.940:0.06922,84489954_Methanosphaera_stadtmanae_DSM_3091_uid58407:0.28465)0.135:0.04696)0.961:0.07329,(20094144_Methanopyrus_kandleri_AV19_uid57883:0.23009,(((337284512_Pyrococcus_yayanosii_CH1_uid68281:0.02189,(18977366_Pyrococcus_furiosus_DSM_3638_uid57873:0.03721,(389852521_Pyrococcus_ST04_uid167261:0.01420,(332158909_Pyrococcus_NA2_uid66551:0.00847,(14590539_Pyrococcus_horikoshii_OT3_uid57753:0.01262,14521589_Pyrococcus_abyssi_GE5_uid62903:0.03986)0.406:0.01088)0.924:0.01829)0.615:0.00858)0.855:0.01635)0.642:0.02351,(57641788_Thermococcus_kodakarensis_KOD1_uid58225:0.06056,(212224826_Thermococcus_onnurineus_NA1_uid59043:0.03383,341582475_Thermococcus_4557_uid70841:0.02891)0.919:0.02978)0.915:0.03226)0.792:0.00954,((223477529_Thermococcus_AM4_uid54735:0.01537,240102255_Thermococcus_gammatolerans_EJ3_uid59389:0.00016)0.977:0.04626,242399930_Thermococcus_sibiricus_MM_739_uid59399:0.11117)0.839:0.02014)0.999:0.13201)0.895:0.05306)0.461:0.08828)1.000:0.30464,(((((432327932_Aciduliprofundum_MAR08_339_uid184407:0.02836,289595684_Aciduliprofundum_boonei_T469_uid43333:0.04621)1.000:0.38214,(488599803_Archaeoglobus_sulfaticallidus_PM70_1_uid201033:0.25231,(490715391_nanoarchaeote_Nst1:0.28219,490715424_nanoarchaeote_Nst1:0.38169)0.996:0.21373)0.589:0.02359)0.778:0.03450,(((432327935_Aciduliprofundum_MAR08_339_uid184407:0.02997,289595687_Aciduliprofundum_boonei_T469_uid43333:0.01373)1.000:0.31394,(11498267_Archaeoglobus_fulgidus_DSM_4304_uid57717:0.22587,((288931899_Ferroglobus_placidus_DSM_10642_uid40863:0.18213,327400022_Archaeoglobus_veneficus_SNP6_uid65269:0.20337)0.819:0.06249,(327400835_Archaeoglobus_veneficus_SNP6_uid65269:0.13735,(288931797_Ferroglobus_placidus_DSM_10642_uid40863:0.13408,284162412_Archaeoglobus_profundus_DSM_5631_uid43493:0.16995)0.296:0.03008)0.950:0.07565)0.792:0.05615)0.987:0.11844)0.882:0.04406,(((((435846523_Natronococcus_occultus_SP4_uid184863:0.10544,(389849140_Haloferax_mediterranei_ATCC_33500_uid167315:0.10553,(((313126821_Halogeometricum_borinquense_DSM_11551_uid54919:0.02355,(345005538_halophilic_archaeon_DL31_uid72619:0.04644,(292655315_Haloferax_volcanii_DS2_uid46845:0.01153,389846633_Haloferax_mediterranei_ATCC_33500_uid167315:0.00014)0.625:0.01625)0.771:0.01500)0.181:0.01198,((433638285_Halovivax_ruber_XH_70_uid184819:0.04377,(((289582785_Natrialba_magadii_ATCC_43099_uid46245:0.01589,(336253957_Halopiger_xanaduensis_SH_6_uid68105:0.00766,397775263_Natrinema_J7_uid171337:0.01939)0.113:0.00780)0.951:0.00014,284164234_Haloterrigena_turkmenica_DSM_5511_uid43501:0.00016)0.808:0.00384,429190527_Natronobacterium_gregoryi_SP2_uid74439:0.00397)0.737:0.00621)0.936:0.02366,((257053920_Halorhabdus_utahensis_DSM_12940_uid59189:0.06803,(257388195_Halomicrobium_mukohataei_DSM_12286_uid59107:0.02883,55378048_Haloarcula_marismortui_ATCC_43049_uid57719:0.00936)0.287:0.01846)0.930:0.02437,76802645_Natronomonas_pharaonis_DSM_2160_uid58435:0.05130)0.853:0.00986)0.858:0.01519)0.060:0.01027,(300711547_Halalkalicoccus_jeotgali_B3_uid50305:0.03853,222479409_Halorubrum_lacusprofundi_ATCC_49239_uid58807:0.06020)0.572:0.01157)0.837:0.02311)0.918:0.04695)1.000:0.22929,(397774918_Natrinema_J7_uid171337:0.19146,292656512_Haloferax_volcanii_DS2_uid46845:0.25669)0.980:0.09628)0.960:0.07717,((((76802193_Natronomonas_pharaonis_DSM_2160_uid58435:0.17515,((510882740_Salinarchaeum_laminariae_Harcht_Bsk1_uid207001:0.10199,(433637758_Halovivax_ruber_XH_70_uid184819:0.08152,(336255092_Halopiger_xanaduensis_SH_6_uid68105:0.05297,284164350_Haloterrigena_turkmenica_DSM_5511_uid43501:0.04322)0.315:0.01670)0.973:0.04684)0.970:0.05470,(15789975_Halobacterium_NRC_1_uid57769:0.15393,((345004659_halophilic_archaeon_DL31_uid72619:0.06141,222479462_Halorubrum_lacusprofundi_ATCC_49239_uid58807:0.05930)0.982:0.06549,(313126951_Halogeometricum_borinquense_DSM_11551_uid54919:0.07317,(389846496_Haloferax_mediterranei_ATCC_33500_uid167315:0.01496,292655193_Haloferax_volcanii_DS2_uid46845:0.04726)0.975:0.04895)0.777:0.03434)0.663:0.02676)0.753:0.01876)0.436:0.00022)0.727:0.02265,(257053875_Halorhabdus_utahensis_DSM_12940_uid59189:0.08463,(55377350_Haloarcula_marismortui_ATCC_43049_uid57719:0.05146,257388761_Halomicrobium_mukohataei_DSM_12286_uid59107:0.04541)0.932:0.04184)0.977:0.04700)1.000:0.19323,(147921247_Methanocella_arvoryzae_MRE50_uid61623:0.20642,((20092769_Methanosarcina_acetivorans_C2A_uid57879:0.03297,21227040_Methanosarcina_mazei_Go1_uid57893:0.00920)1.000:0.15837,((20089132_Methanosarcina_acetivorans_C2A_uid57879:0.03163,21227618_Methanosarcina_mazei_Go1_uid57893:0.02289)1.000:0.29796,((294496202_Methanohalophilus_mahii_DSM_5219_uid47313:0.14329,91774309_Methanococcoides_burtonii_DSM_6242_uid58023:0.13433)0.950:0.05635,(298675314_Methanohalobium_evestigatum_Z_7303_uid49857:0.14977,410672357_Methanolobus_psychrophilus_R15_uid177925:0.12140)0.824:0.01920)0.921:0.04096)0.609:0.02967)0.990:0.09639)0.874:0.07049)0.889:0.03068,((((327401123_Archaeoglobus_veneficus_SNP6_uid65269:0.07482,(((11497950_Archaeoglobus_fulgidus_DSM_4304_uid57717:0.04746,11498601_Archaeoglobus_fulgidus_DSM_4304_uid57717:0.02145)0.990:0.06042,(284161835_Archaeoglobus_profundus_DSM_5631_uid43493:0.08712,488600976_Archaeoglobus_sulfaticallidus_PM70_1_uid201033:0.04714)0.986:0.05142)0.578:0.01361,(288930649_Ferroglobus_placidus_DSM_10642_uid40863:0.01170,288931167_Ferroglobus_placidus_DSM_10642_uid40863:0.00014)1.000:0.09191)0.827:0.01560)0.839:0.02234,327401535_Archaeoglobus_veneficus_SNP6_uid65269:0.06900)0.992:0.07637,((282163997_Methanocella_paludicola_SANAE_uid42887:0.04379,147919598_Methanocella_arvoryzae_MRE50_uid61623:0.05155)0.997:0.09733,(298674470_Methanohalobium_evestigatum_Z_7303_uid49857:0.09122,(336476396_Methanosalsum_zhilinae_DSM_4017_uid68249:0.11579,(410669621_Methanolobus_psychrophilus_R15_uid177925:0.07264,(294495978_Methanohalophilus_mahii_DSM_5219_uid47313:0.06113,91772493_Methanococcoides_burtonii_DSM_6242_uid58023:0.08160)0.929:0.03556)0.879:0.01750)0.688:0.01664)0.975:0.06980)0.991:0.08546)0.888:0.03092,(((307353958_Methanoplanus_petrolearius_DSM_11571_uid52695:0.09171,(397779428_Methanoculleus_bourgensis_MS2_uid171377:0.01785,126179946_Methanoculleus_marisnigri_JR1_uid58561:0.01736)0.993:0.06568)0.806:0.01638,((432330483_Methanoregula_formicicum_SMSP_uid184406:0.01547,(154151043_Methanoregula_boonei_6A8_uid58815:0.07528,154151446_Methanoregula_boonei_6A8_uid58815:0.00716)0.380:0.01597)1.000:0.09801,88604393_Methanospirillum_hungatei_JF_1_uid58181:0.10829)0.264:0.01395)0.999:0.16162,(336476402_Methanosalsum_zhilinae_DSM_4017_uid68249:0.25930,(91772319_Methanococcoides_burtonii_DSM_6242_uid58023:0.11886,(410670047_Methanolobus_psychrophilus_R15_uid177925:0.12330,435851879_Methanomethylovorans_hollandica_DSM_15978_uid184864:0.09068)0.608:0.05025)0.753:0.04726)0.996:0.13543)0.766:0.04522)0.836:0.04930)0.849:0.02368)0.922:0.03318,(124486275_Methanocorpusculum_labreanum_Z_uid58785:0.40071,(88602461_Methanospirillum_hungatei_JF_1_uid58181:0.11890,(307352384_Methanoplanus_petrolearius_DSM_11571_uid52695:0.16155,(397779327_Methanoculleus_bourgensis_MS2_uid171377:0.12945,((432329743_Methanoregula_formicicum_SMSP_uid184406:0.08132,154151894_Methanoregula_boonei_6A8_uid58815:0.08543)0.989:0.07569,219853242_Methanosphaerula_palustris_E1_9c_uid59193:0.07890)0.757:0.03049)0.000:0.03445)0.610:0.03882)0.960:0.08187)0.989:0.11544)0.886:0.03725,(((154151925_Methanoregula_boonei_6A8_uid58815:0.12637,432330325_Methanoregula_formicicum_SMSP_uid184406:0.15136)0.996:0.12300,((124486352_Methanocorpusculum_labreanum_Z_uid58785:0.22316,(307352530_Methanoplanus_petrolearius_DSM_11571_uid52695:0.15318,307354364_Methanoplanus_petrolearius_DSM_11571_uid52695:0.14357)0.417:0.03797)0.415:0.05212,(307352780_Methanoplanus_petrolearius_DSM_11571_uid52695:0.12698,88601625_Methanospirillum_hungatei_JF_1_uid58181:0.27986)0.265:0.04172)0.972:0.09454)0.999:0.16651,(288930966_Ferroglobus_placidus_DSM_10642_uid40863:0.35989,(282165242_Methanocella_paludicola_SANAE_uid42887:0.14808,147920828_Methanocella_arvoryzae_MRE50_uid61623:0.12587)1.000:0.52851)0.884:0.09275)0.337:0.05636)0.946:0.05609)0.901:0.03839)0.935:0.06181,((407462573_Candidatus_Nitrosopumilus_koreensis_AR1_uid176129:0.00016,(340344981_Nitrosoarchaeum_koreensis_MY1_MY1:0.04359,407464921_Candidatus_Nitrosopumilus_AR2_uid176130:0.01151)0.887:0.02253)1.000:0.36014,((154151961_Methanoregula_boonei_6A8_uid58815:0.13918,307354776_Methanoplanus_petrolearius_DSM_11571_uid52695:0.14176)0.977:0.10729,(20091914_Methanosarcina_acetivorans_C2A_uid57879:0.11299,410669581_Methanolobus_psychrophilus_R15_uid177925:0.14227)0.966:0.10457)1.000:0.29979)0.882:0.07832)0.332:0.03228,((((((((488600380_Archaeoglobus_sulfaticallidus_PM70_1_uid201033:0.05084,(284162484_Archaeoglobus_profundus_DSM_5631_uid43493:0.01951,11498654_Archaeoglobus_fulgidus_DSM_4304_uid57717:0.02839)0.973:0.03714)0.281:0.02761,(91772405_Methanococcoides_burtonii_DSM_6242_uid58023:0.11354,(20091874_Methanosarcina_acetivorans_C2A_uid57879:0.01128,21226419_Methanosarcina_mazei_Go1_uid57893:0.00020)1.000:0.10114)0.960:0.06650)0.705:0.06045,(88601423_Methanospirillum_hungatei_JF_1_uid58181:0.15349,(307354195_Methanoplanus_petrolearius_DSM_11571_uid52695:0.09646,((397781053_Methanoculleus_bourgensis_MS2_uid171377:0.02394,126178899_Methanoculleus_marisnigri_JR1_uid58561:0.02630)0.989:0.05437,(219852458_Methanosphaerula_palustris_E1_9c_uid59193:0.08566,(154150885_Methanoregula_boonei_6A8_uid58815:0.01972,432330910_Methanoregula_formicicum_SMSP_uid184406:0.02822)0.982:0.05570)0.835:0.01657)0.925:0.03651)0.751:0.03599)0.998:0.14661)0.776:0.04986,(147921416_Methanocella_arvoryzae_MRE50_uid61623:0.14800,(20091900_Methanosarcina_acetivorans_C2A_uid57879:0.02994,21226515_Methanosarcina_mazei_Go1_uid57893:0.00015)1.000:0.21154)0.605:0.04897)0.996:0.10686,(305664035_Ignisphaera_aggregans_DSM_17230_uid51875:0.09622,(296242934_Thermosphaera_aggregans_DSM_11486_uid48993:0.09587,((549455838_Aeropyrum_camini_SY1___JCM_12091_uid222311:0.00071,118431640_Aeropyrum_pernix_K1_uid57757:0.01086)0.994:0.08558,(385805971_Fervidicoccus_fontis_Kam940_uid162201:0.08598,302349263_Acidilobus_saccharovorans_345_15_uid51395:0.18333)0.189:0.03346)0.378:0.04024)0.989:0.10357)0.989:0.09514)0.415:0.00926,((490715305_nanoarchaeote_Nst1:0.32211,((327401350_Archaeoglobus_veneficus_SNP6_uid65269:0.22153,((313126764_Halogeometricum_borinquense_DSM_11551_uid54919:0.01329,(389846688_Haloferax_mediterranei_ATCC_33500_uid167315:0.00498,292655371_Haloferax_volcanii_DS2_uid46845:0.00291)0.972:0.03589)0.898:0.02954,(((510882471_Salinarchaeum_laminariae_Harcht_Bsk1_uid207001:0.05165,((433637567_Halovivax_ruber_XH_70_uid184819:0.01340,(435847644_Natronococcus_occultus_SP4_uid184863:0.01917,284166669_Haloterrigena_turkmenica_DSM_5511_uid43501:0.02693)0.916:0.02858)0.999:0.07174,(15790072_Halobacterium_NRC_1_uid57769:0.0,169235794_Halobacterium_salinarum_R1_uid61571:0.0):0.04285)0.822:0.01701)0.749:0.01903,((76801723_Natronomonas_pharaonis_DSM_2160_uid58435:0.02441,(429191904_Natronobacterium_gregoryi_SP2_uid74439:0.01871,(336254902_Halopiger_xanaduensis_SH_6_uid68105:0.02549,289582523_Natrialba_magadii_ATCC_43099_uid46245:0.00889)0.558:0.01031)0.942:0.01918)0.970:0.03896,(55378881_Haloarcula_marismortui_ATCC_43049_uid57719:0.05928,222480973_Halorubrum_lacusprofundi_ATCC_49239_uid58807:0.04470)0.774:0.02047)0.849:0.01764)0.828:0.01393,(257052043_Halorhabdus_utahensis_DSM_12940_uid59189:0.05797,257386301_Halomicrobium_mukohataei_DSM_12286_uid59107:0.04800)0.771:0.00865)0.947:0.05494)1.000:0.22033)0.970:0.08879,((((288931828_Ferroglobus_placidus_DSM_10642_uid40863:0.17165,(((296109772_Methanocaldococcus_infernus_ME_uid48803:0.01672,(256811146_Methanocaldococcus_fervens_AG86_uid59347:0.02642,15669090_Methanocaldococcus_jannaschii_DSM_2661_uid57713:0.01687)0.872:0.01541)0.951:0.04520,(336121892_Methanothermococcus_okinawensis_IH1_uid51535:0.03654,(150400702_Methanococcus_aeolicus_Nankai_3_uid58823:0.10136,(297619812_Methanococcus_voltae_A3_uid49529:0.05595,45359238_Methanococcus_maripaludis_S2_uid58035:0.02820)0.834:0.01672)0.919:0.01777)0.964:0.04312)0.982:0.08546,(298675222_Methanohalobium_evestigatum_Z_7303_uid49857:0.10258,(336477577_Methanosalsum_zhilinae_DSM_4017_uid68249:0.01624,(410671551_Methanolobus_psychrophilus_R15_uid177925:0.02448,(435850688_Methanomethylovorans_hollandica_DSM_15978_uid184864:0.05981,91773537_Methanococcoides_burtonii_DSM_6242_uid58023:0.04733)0.781:0.02706)0.995:0.06618)0.945:0.04919)1.000:0.19658)0.460:0.05141)0.294:0.03456,(242398767_Thermococcus_sibiricus_MM_739_uid59399:0.10883,((223478325_Thermococcus_AM4_uid54735:0.00725,(212224328_Thermococcus_onnurineus_NA1_uid59043:0.00333,57639983_Thermococcus_kodakarensis_KOD1_uid58225:0.02659)0.968:0.02349)0.979:0.03933,(530548644_Thermococcus_litoralis_DSM_5473_uid82997:0.02035,((14521686_Pyrococcus_abyssi_GE5_uid62903:0.01880,14590457_Pyrococcus_horikoshii_OT3_uid57753:0.00374)0.945:0.01805,(18976703_Pyrococcus_furiosus_DSM_3638_uid57873:0.01597,337284711_Pyrococcus_yayanosii_CH1_uid68281:0.01444)0.306:0.01842)0.979:0.03455)0.700:0.00320)0.634:0.01856)0.975:0.06265)0.874:0.05382,(13541444_Thermoplasma_volcanium_GSS1_uid57751:0.02185,16081663_Thermoplasma_acidophilum_DSM_1728_uid61573:0.00432)1.000:0.21095)0.405:0.03360,(432329155_Aciduliprofundum_MAR08_339_uid184407:0.02080,289596992_Aciduliprofundum_boonei_T469_uid43333:0.00569)1.000:0.17896)0.836:0.02913)0.950:0.07000)0.984:0.09933,(408403630_Candidatus_Nitrososphaera_gargensis_Ga9_2_uid176707:0.33220,(((15922858_Sulfolobus_tokodaii_7_uid57807:0.02500,146304091_Metallosphaera_sedula_DSM_5348_uid58717:0.08811)0.829:0.02312,(227829136_Sulfolobus_islandicus_L_S_2_15_uid58871:0.01342,15899076_Sulfolobus_solfataricus_P2_uid57721:0.00646)0.964:0.03194)0.048:0.00809,70606943_Sulfolobus_acidocaldarius_DSM_639_uid58379:0.05647)1.000:0.25614)0.848:0.04585)0.857:0.03910)0.889:0.03962,557694873_Candidatus_Caldiarchaeum_subterraneum_uid227223:0.23421)1.000:0.23311,(256810063_Methanocaldococcus_fervens_AG86_uid59347:0.37051,(41614965_Nanoarchaeum_equitans_Kin4_M_uid58009:0.24649,490715254_nanoarchaeote_Nst1:0.12133)0.998:0.18099)0.514:0.06112)0.740:0.05050)0.901:0.03361)0.902:0.04854)0.990:0.10685,((((385805133_Fervidicoccus_fontis_Kam940_uid162201:0.36369,(320100682_Desulfurococcus_mucosus_DSM_2162_uid62227:0.13033,(390938538_Desulfurococcus_fermentans_DSM_16532_uid75119:0.00786,218884009_Desulfurococcus_kamchatkensis_1221n_uid59133:0.00941)0.993:0.13952)0.755:0.18019)1.000:0.89982,(((352682167_Thermoproteus_tenax_Kra_1_uid74443:0.18557,327310537_Thermoproteus_uzoniensis_768_20_uid65089:0.09703)0.984:0.33666,(119872380_Pyrobaculum_islandicum_DSM_4184_uid58635:0.13256,(126458953_Pyrobaculum_calidifontis_JCM_11548_uid58787:0.07979,18313317_Pyrobaculum_aerophilum_IM2_uid57727:0.09886)0.567:0.05283)0.853:0.13441)1.000:1.53749,(((325969018_Vulcanisaeta_moutnovskia_768_28_uid63631:0.11650,307594784_Vulcanisaeta_distributa_DSM_14429_uid52827:0.04195)1.000:0.97571,(159041458_Caldivirga_maquilingensis_IC_167_uid58711:0.66899,(307596359_Vulcanisaeta_distributa_DSM_14429_uid52827:0.23963,(325968523_Vulcanisaeta_moutnovskia_768_28_uid63631:0.06544,307594291_Vulcanisaeta_distributa_DSM_14429_uid52827:0.10221)0.568:0.09303)1.000:0.53153)0.987:0.33298)0.613:0.12733,435849750_Methanomethylovorans_hollandica_DSM_15978_uid184864:2.17451)0.566:0.12117)0.899:0.10201)0.699:0.03052,(((41615211_Nanoarchaeum_equitans_Kin4_M_uid58009:0.41140,490715604_nanoarchaeote_Nst1:0.29565)0.966:0.23725,((478482923_archaeon_Mx1201_uid196597:0.08805,474934545_Thermoplasmatales_archaeon_BRNA1_uid195930:0.08683)1.000:0.61311,((((222478633_Halorubrum_lacusprofundi_ATCC_49239_uid58807:0.33819,(345004072_halophilic_archaeon_DL31_uid72619:0.39110,313127198_Halogeometricum_borinquense_DSM_11551_uid54919:0.19043)0.000:0.04989)0.785:0.05250,(((257389096_Halomicrobium_mukohataei_DSM_12286_uid59107:0.34243,(76803230_Natronomonas_pharaonis_DSM_2160_uid58435:0.31964,510882164_Salinarchaeum_laminariae_Harcht_Bsk1_uid207001:0.25489)0.228:0.04934)0.454:0.06955,((429191319_Natronobacterium_gregoryi_SP2_uid74439:0.11769,284176147_Haloterrigena_turkmenica_DSM_5511_uid43501:0.16403)0.559:0.04657,(336254332_Halopiger_xanaduensis_SH_6_uid68105:0.12674,257051105_Halorhabdus_utahensis_DSM_12940_uid59189:0.19689)0.666:0.03671)1.000:0.19256)0.941:0.08155,(389846176_Haloferax_mediterranei_ATCC_33500_uid167315:0.07398,292654911_Haloferax_volcanii_DS2_uid46845:0.07874)1.000:0.18337)0.500:0.04092)0.640:0.08302,110667163_Haloquadratum_walsbyi_DSM_16790_uid58673:0.34573)1.000:0.42208,((282165642_Methanocella_paludicola_SANAE_uid42887:0.12131,147920585_Methanocella_arvoryzae_MRE50_uid61623:0.15485)0.988:0.19197,(336477115_Methanosalsum_zhilinae_DSM_4017_uid68249:0.12750,(91772127_Methanococcoides_burtonii_DSM_6242_uid58023:0.07759,(435852098_Methanomethylovorans_hollandica_DSM_15978_uid184864:0.19272,(20090163_Methanosarcina_acetivorans_C2A_uid57879:0.05199,21228400_Methanosarcina_mazei_Go1_uid57893:0.01568)1.000:0.18926)0.707:0.04242)0.953:0.07186)0.999:0.27135)0.982:0.19006)0.790:0.08038)0.611:0.09322)1.000:0.69752,(((((((76800735_Natronomonas_pharaonis_DSM_2160_uid58435:0.19037,(55378675_Haloarcula_marismortui_ATCC_43049_uid57719:0.06722,257386198_Halomicrobium_mukohataei_DSM_12286_uid59107:0.15808)0.881:0.05861)0.804:0.04404,510881457_Salinarchaeum_laminariae_Harcht_Bsk1_uid207001:0.12013)0.847:0.02841,((389846072_Haloferax_mediterranei_ATCC_33500_uid167315:0.00016,292654785_Haloferax_volcanii_DS2_uid46845:0.03508)1.000:0.14071,(110667293_Haloquadratum_walsbyi_DSM_16790_uid58673:0.13880,313127311_Halogeometricum_borinquense_DSM_11551_uid54919:0.04929)0.934:0.03363)0.518:0.02115)0.732:0.02774,222478655_Halorubrum_lacusprofundi_ATCC_49239_uid58807:0.15140)0.814:0.05382,345006272_halophilic_archaeon_DL31_uid72619:0.10004)1.000:0.81614,(170290933_Candidatus_Korarchaeum_cryptofilum_OPF8_uid58601:1.64915,((325967821_Vulcanisaeta_moutnovskia_768_28_uid63631:0.14736,307595966_Vulcanisaeta_distributa_DSM_14429_uid52827:0.16295)0.997:0.58869,((126459667_Pyrobaculum_calidifontis_JCM_11548_uid58787:0.27966,(119872468_Pyrobaculum_islandicum_DSM_4184_uid58635:0.35770,(145592329_Pyrobaculum_arsenaticum_DSM_13514_uid58409:0.24959,18313110_Pyrobaculum_aerophilum_IM2_uid57727:0.28965)0.197:0.06835)0.811:0.08987)0.990:0.34124,(352683097_Thermoproteus_tenax_Kra_1_uid74443:0.43853,327311453_Thermoproteus_uzoniensis_768_20_uid65089:0.45426)0.948:0.25019)0.994:0.47637)0.993:0.49369)0.850:0.16192)0.886:0.16919,(13541846_Thermoplasma_volcanium_GSS1_uid57751:0.40272,16081953_Thermoplasma_acidophilum_DSM_1728_uid61573:0.38427)1.000:0.54168)0.650:0.07068)0.700:0.06857)0.910:0.04470,((530780550_Thermofilum_1910b_uid215374:0.22922,119719637_Thermofilum_pendens_Hrk_5_uid58563:0.45147)1.000:1.58070,(332796255_Acidianus_hospitalis_W1_uid66875:3.77755,(13541843_Thermoplasma_volcanium_GSS1_uid57751:0.59344,16081950_Thermoplasma_acidophilum_DSM_1728_uid61573:0.64428)0.849:0.33801)0.935:0.42597)0.810:0.18206)0.184:0.03302);
