# Supplementary material for: Diversity and Evolution of Type IV pili Systems in Archaea
Source: Front Microbiol. 2016 May 6;7:667. doi: 10.3389/fmicb.2016.00667 (PMC4858521; doi:10.3389/fmicb.2016.00667)
Supplement: Supplementary file 1 [file Presentation_1.ZIP › Supplementary_Table_S6.docx]

**Table S6. Sequence similarity search results for selected proteins**

| arCOG | Query GI | Program* | Hit ID and annotation | HHpred probablity (Pr) or PSI-BLAST  E-value (E) | Comment (I-iteration) |
| --- | --- | --- | --- | --- | --- |
| arCOG02981 | 147920583 | PSI_BLAST | flagellin [Halostagnicola larsenii XH-48] GI:573482015 | E=5e-06 | I=2; ”KG” motif followed by SP |
| arCOG10216 | 504368010 | HHpred | TIGR01710 typeII_sec_gspG type II secretion system protein G | Pr=52.65 | ”R” motif followed by SP; HHpred also finds other hits to pilins (mostly of type II systems) |
| arCOG10359 | 307594292 | PSI_BLAST | type II secretion system F domain-containing protein [Vulcanisaeta moutnovskia 768-28] GI: 325969263 | E=0.006 | I=2; |
| arCOG10434 | 307594294 | HHpred | PF01917 Arch_flagellin | Pr=52.87 | Probably closer to FlaG |
| arCOG13767 | 503369718 | HHpred | 2hi2_A Fimbrial protein; type IV pilin | Pr=93.19 | Many hits to other pilins |
| arCOG08521 | 15899406 | HHpred | TIGR02537 arch_flag_Nterm archaeal flagellin N-terminal-like domain | Pr=84.47 | FlaG/FlaF subfamily |
| arCOG08521 | 15899405 | HHpred | COG3353 FlaF Putative archaeal flagellar protein F | Pr=76.03 | FlaG/FlaF subfamily |
| arCOG08836 | 126466361 | PSI_BLAST | flagellin [Thermosphaera aggregans] GI: 502894330 | E=0.002 | I=2; same as arCOG07276 ”R[GA] motif |
| arCOG02911 | 300711541 | HHpred | TIGR02537 arch_flag_Nterm archaeal flagellin N-terminal-like domain | Pr=96.86 | Distinct conserved pilin associated mostly with euryarchaeal systems |
| arCOG07206 | 70606947 | HHpred | pfam05377 FlaC_arch Flagella accessory protein C (FlaC) | Pr=83.20 |  |
| arCOG03739 | 18312267 | PSI_BLAST | hypothetical protein SacRon12I_11535 [Sulfolobus acidocaldarius  Ron12/I] GI: 449070588 | E= 8e-05 | I=2. SacRon12I_11535 belongs to arCOG03871 (major pilin) |

PSI_BLAST searches were run with the following parameters: inclusion treshold = 0.01, nt dtabase restriction: ”archaea” only,

No low complexity filtering, composition based statistics – off, unless stated otherwise in comments

HHpred – defalt parameters with PSI-BLAST interation = 3, against pdb and cdd databases
