# Supplementary material for: Diversity and Evolution of Type IV pili Systems in Archaea
Source: Front Microbiol. 2016 May 6;7:667. doi: 10.3389/fmicb.2016.00667 (PMC4858521; doi:10.3389/fmicb.2016.00667)
Supplement: Supplementary file 1 [file Presentation_1.ZIP › Supplementary_Table_S5.docx]

**Table S5. Additional arCOGs that might be structural or regulatory components of respective T4P in some organisms but not in others**

| arCOG | GI | Comment |
| --- | --- | --- |
| **arCOGs that are present in VirB11 neighborhoods** | | |
| arCOG02079 | 340344977 | Predicted S-layer protein, linked to the membrane |
| arCOG02493 | 282165522 | Predicted surface protein, Flafind positive |
| arCOG02508 | 15669659 | Large surface protein with PKD repeats |
| arCOG02945 | 282165621 | Surface protein |
| arCOG03269 | 336476494 | Surface protein, contains DUF11 domain, possibly involved in archaellum system in methanomicrobia |
| arCOG03508 | 15669662 | Predicted S-layer protein, associated with branch 1 pili system |
| arCOG03512 | 332159558 | Predicted S-layer protein, associated with branch 1 pili system |
| arCOG03359 | 435850781 | Specific for Methanomicrobia; Secreted alpha + beta protein |
| arCOG04995 | 11499052 | Small membrane protein similar to arCOG06518, likely assicated with archaellum |
| arCOG05173 | 282165622 | Surface protein with PAF C-terminal sorting motif |
| arCOG05607 | 126458955 | Specific for Pyrobaculum group, small, mostly alpha helical cytoplasmic protein |
| arCOG05605 | 126458957 | Specific for Pyrobaculum group. Alpha/beta cytoplasmic protein |
| arCOG05606 | 126458956 | Specific for Pyrobaculum group, alpha helical cytoplasmic protein |
| arCOG05610 | 126458951 | Specific for Pyrobaculum group, alpha helical cytoplasmic protein |
| arCOG05762 | 18977984 | Specific for Thermococci secreted alpha-helical protein, linked to FleN-like ATPase from arCOG00589 |
| arCOG06273 | 389847892 | Specific for Halobacteria; predicted S-layer protein; Many paralogs |
| arCOG06944 | 55380047 | Specific for Halobacteria/Methanomicrobia; Membrane protein (12-16, rarely 4 TMs) |
| arCOG06945 | 119718972 | Small secreted protein, specific for Thermofilum, but also present in Nanoarchaeum equitans where it is not linked to T4P system |
| arCOG07264 | 15899407 | 5 TM membrane protein with C-terminal Zn ribbon domain; Specific for binosome loci (subclade 4I) of Sulfolobales lineage |
| arCOG07329 | 15899400 | 8 TM membrane protein; Specific for binosome loci (subclade 4I) of Sulfolobales lineage |
| arCOG07479 | 18313318 | Specific for Pyrobaculum group, small alpha helical cytoplasmic protein |
| arCOG07862 | 147921092 | Uncharacterized protein containing PA14 domain, FlaFind positive and encoded next to pilins in Methanocella, but in other archaea and bacteria rarely assocaited with T4P systems |
| arCOG09300 | 15791008\| | Specific for Halobacterium genus, Small membrane protein |
| arCOG10833 | 336476501 | Membrane protein, possibly involved in archaellum system in methanomicrobia |
| arCOG12061 | 282162762 | Predicted surface protein more often associated with S-layer like protein, which in turn often found in T4P context |
| arCOG13816 | 307595032 | ”R” motif followed by SP, possible component in Vulcanisaeta species |
| arCOG14668 | 530780774 | Large secreted protein, specific for Thermofilum |
| **arCOGs similar to components of archaeal T4P systems, which are not present in the respective operons** | | |
| arCOG02945 | 282165621 | Surface protein |
| arCOG03359 | 435850781 | Specific for Methanomicrobia; Secreted alpha + beta protein |
| arCOG04995 | 11499052 | Small membrane protein |
| arCOG05173 | 282165622 | Surface protein with PAF C-terminal sorting motif |
| arCOG05607 | 126458955 | Specific for Pyrobaculum group, small, mostly alpha helical cytoplasmic protein |
| arCOG05605 | 126458957 | Specific for Pyrobaculum group. Alpha/beta cytoplasmic protein |
| arCOG05606 | 126458956 | Specific for Pyrobaculum group, alpha helical cytoplasmic protein |
| arCOG06273 | 389847892 | Specific for Halobacteria; predicted S-layer protein; Many paralogs |
| arCOG06944 | 55380047 | Specific for Halobacteria/Methanomicrobia; Membrane protein (12-16, rarely 4 TMs) |
| arCOG09300 | 15791008\| | Specific for Halobacterium genus, Small membrane protein |
| arCOG15189  and  arCOG15198 | 76800829 | Small proteins with predicted 2 membrane domains |
