# Supplementary material for: Diversity and Evolution of Type IV pili Systems in Archaea
Source: Front Microbiol. 2016 May 6;7:667. doi: 10.3389/fmicb.2016.00667 (PMC4858521; doi:10.3389/fmicb.2016.00667)
Supplement: Supplementary file 1 [file Presentation_1.ZIP › Supplementary_Figures_Legends.docx]

**Supplementary Figure S1. Clade 1 T4P loci organization**

Gene loci predicted to be associated with this systems are shown for selected representative genomes. Only one gene (or a tandem) for stand alone major pilin is shown (since there could be several stand alone pilin genes in each genome). The genes are represented by arrows. The scale of the arrow is roughly proportional to gene length. The arrow indicates direction of gene. The red triangles above a gene denote genes that encode FlaFind positive arCOG members. The blue triangles above a gene denote genes that encode proteins predicted to be secreted. Small blue square with a number inside indicates a protein with corresponding number of TM domains. Under the genes arCOG number is indicated (if it is assigned to an arCOG). Coloring of the archaeal genomes in the tree is the same as in the Figure 1. Color code for the genes is the following: secretion ATPases – orange, FleN/MinD family ATPase – bright yellow, TadC-like proteins – brown, major and minor pilin – magenta, adhesins – purple, prepilin signal peptidase – green; S-layer-like proteins are dark blue. Genes with no prediction of function but possible components of the system are shown by white arrows with red outline; genes that have some meaningful annotation but not related to T4P system, which can be regulatory components or components that co-translated with system as part of large regulon are shown by gray arrows with red outline. Genes that are unlikely components of the system are shown by gray arrows with black outline.

**Supplementary Figure S2. Archaellum loci organization**

Color scheme is the same as on the Figure 3. Additional color coding corresponds to the genes shown in the typical archaellum operon in the panel B.

**Supplementary Figure S3. Clade 2 T4P loci organization**

Designations and color coding is the same as on the Figure 2.

**Supplementary Figure S4. Domain organization of secretion ATPases**

**Supplementary Figure S5. Subclade 4C and 4F T4P loci organization**

1. **Subclade 4C loci organization**
2. **Subclade 4F loci organization**

Designations and color coding is the same as on the Figure 2.

**Supplementary Figure S6. Subclade 4A, 4I, 4H and 4J T4P loci organization**

1. **Subclade 4A loci organization**
2. **Subclade 4I loci organization**
3. **Subclade 4H loci organization**
4. **Subclade 4J loci organization**

Designations and color coding is the same as on the Figure 2.

**Supplementary Figure S7. Subclade 4B, 4D, 4E and 4G T4P loci organization**

1. **Subclade 4B loci organization**
2. **Subclade 4D loci organization**
3. **Subclade 4E loci organization**
4. **Subclade 4G loci organization**

Designations and color coding is the same as on the Figure 2. In the panel C the magenta dashed lines connect homologous genes in the two small branches outside of the described subclades of clade 4.

**Supplementary Table S1. List and the respective phyletic profiles for all arCOGs found to be associated with archaeal T4P systems.**

**Supplementary Table S2. List of all secretion ATPases found in archaeal genomes and assignment them to clades according to phylogenetic tree.**

**Supplementary Table S3. Presence of secretion ATPases from four major clades in archaeal genomes.**

**Supplementary Table S4. Complete list of all genomic loci containing arCOGs associated with archaeal T4P systems.**

**Supplementary Table 5. Additional arCOGs that might be structural or regulatory components of respective T4P in some organisms but not in others.**

**Supplementary Table S6. Sequence similarity search results for selected proteins.**
