# Supplementary figures and images for: Diversity and Evolution of Type IV pili Systems in Archaea
Source: Front Microbiol. 2016 May 6;7:667. doi: 10.3389/fmicb.2016.00667 (PMC4858521; doi:10.3389/fmicb.2016.00667)

**B**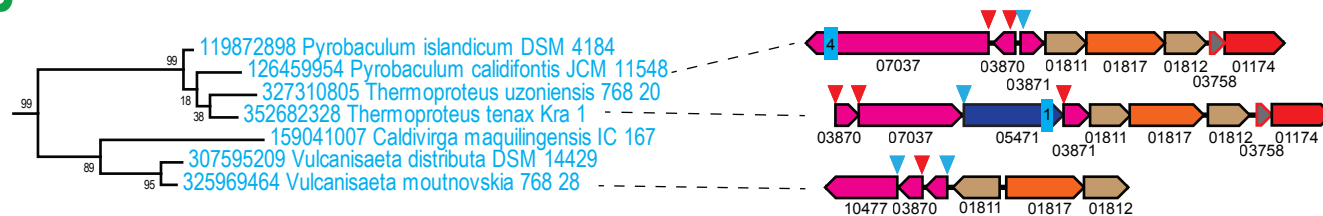**D**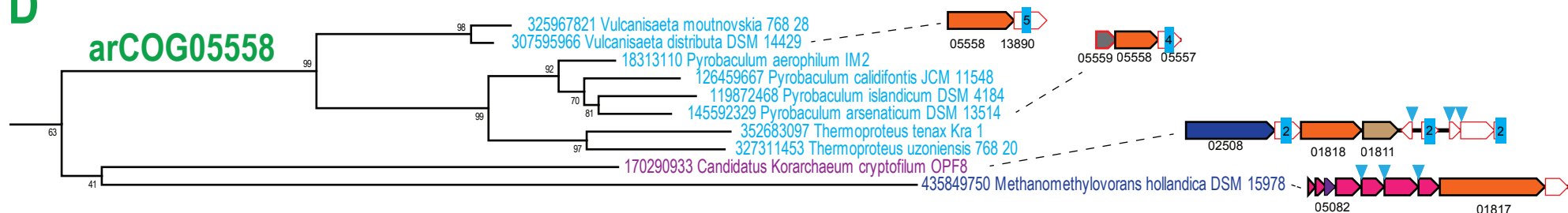**E**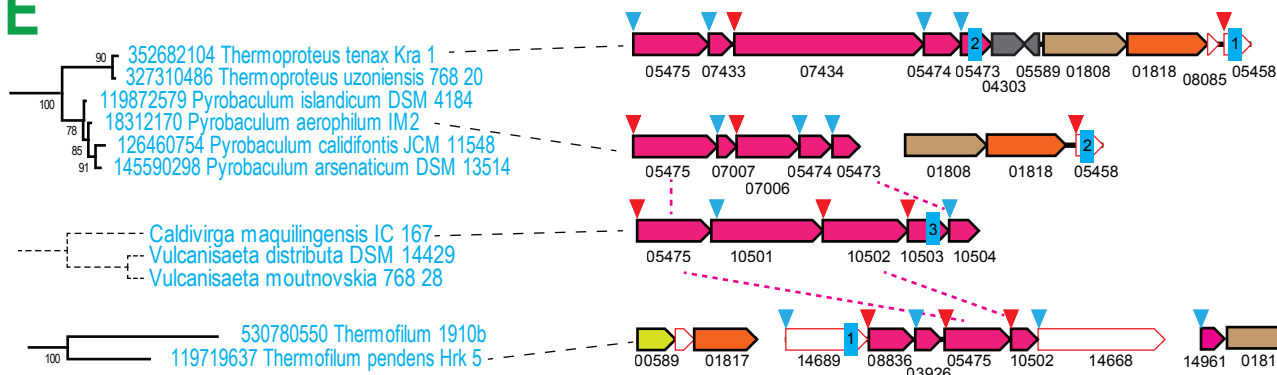**G**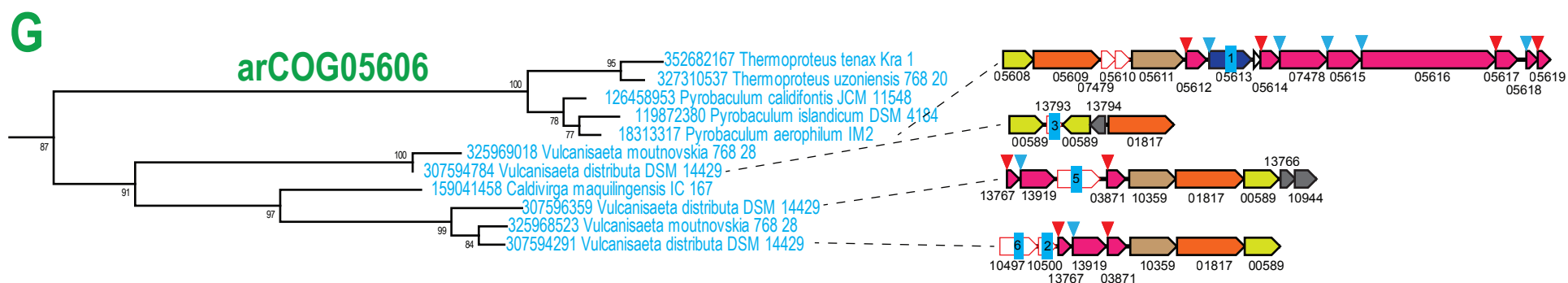

Supplementary Figure S7

Supplement: Supplementary file 1 [file Presentation_1.ZIP › makarova_Frontiers_Figure_S7.pdf]

A

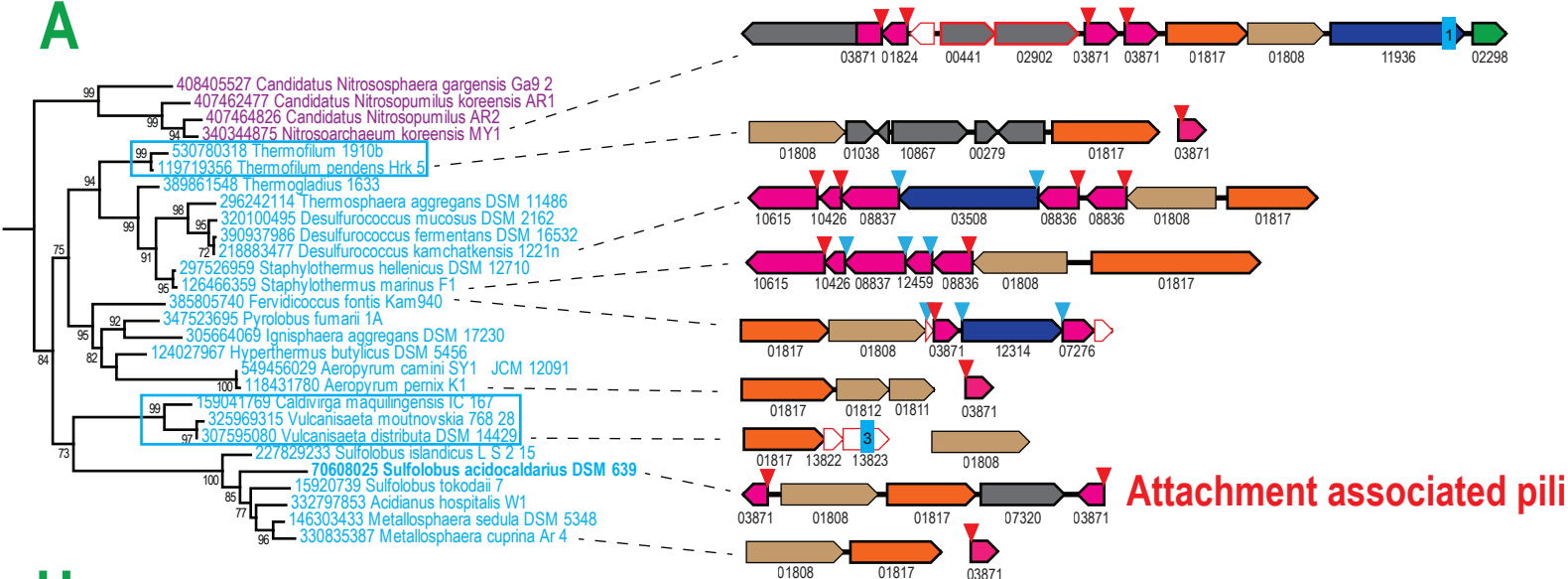

H

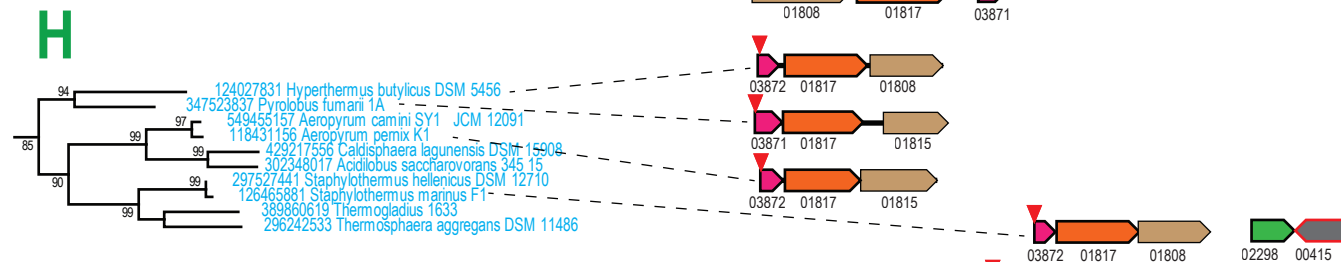

I

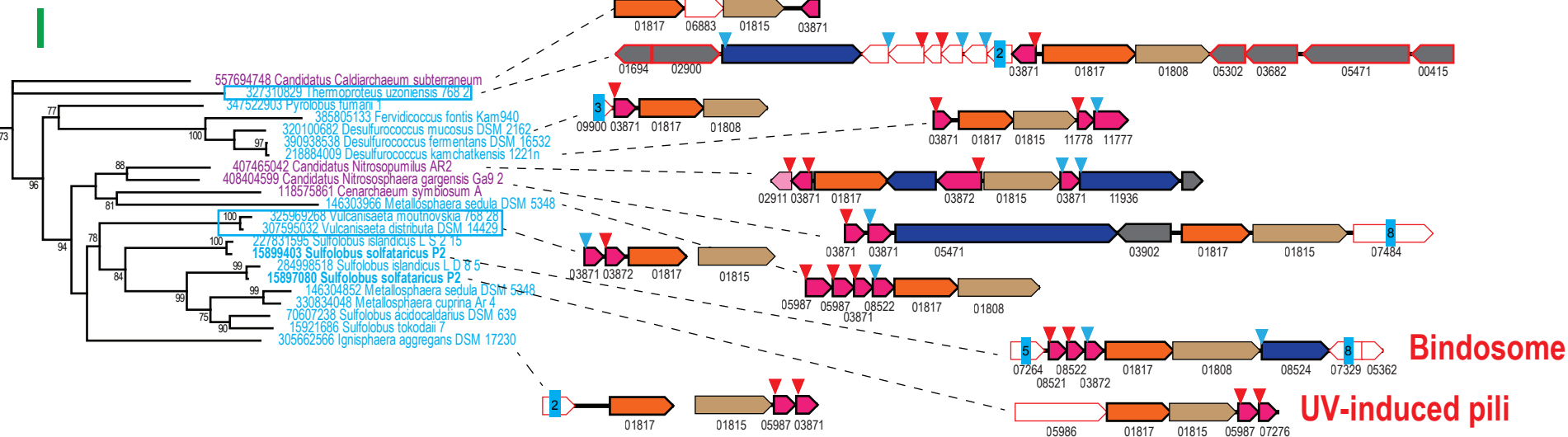

J

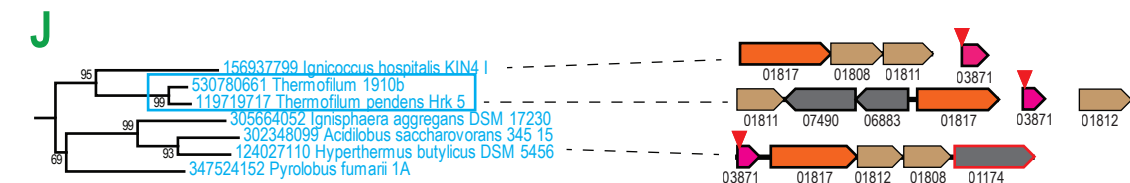

Supplement: Supplementary file 1 [file Presentation_1.ZIP › makarova_Frontiers_Figure_S6.pdf]

C

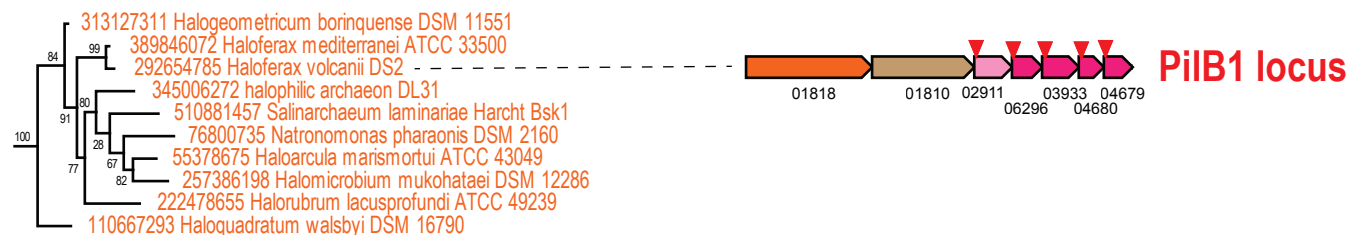

F

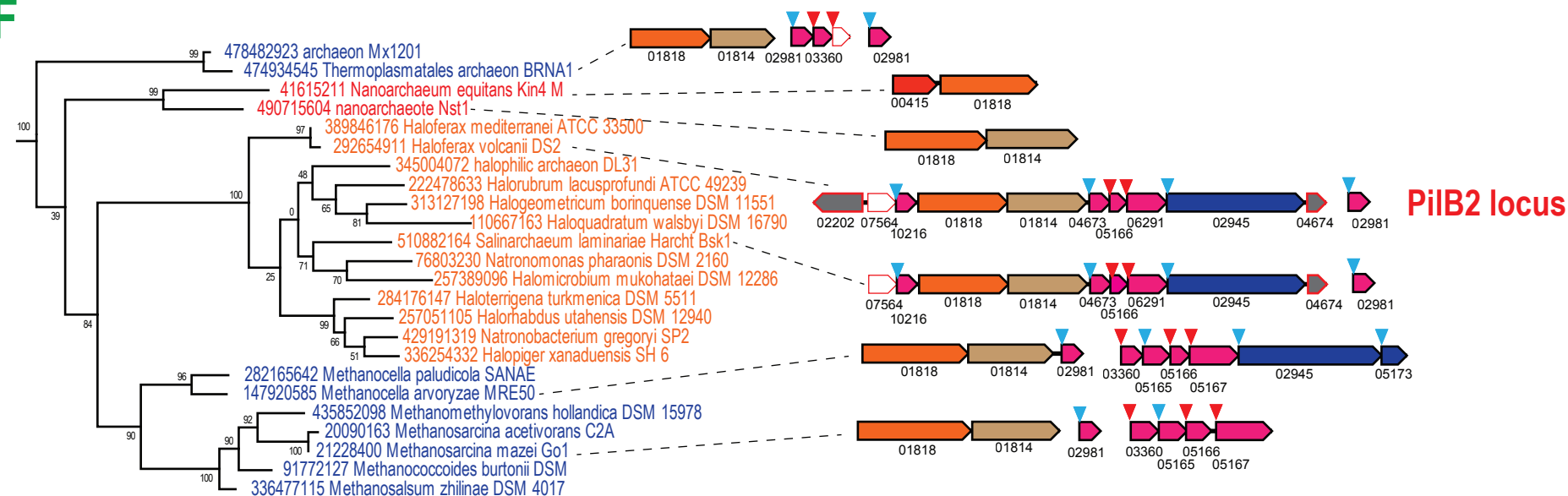

Supplement: Supplementary file 1 [file Presentation_1.ZIP › makarova_Frontiers_Figure_S5.pdf]

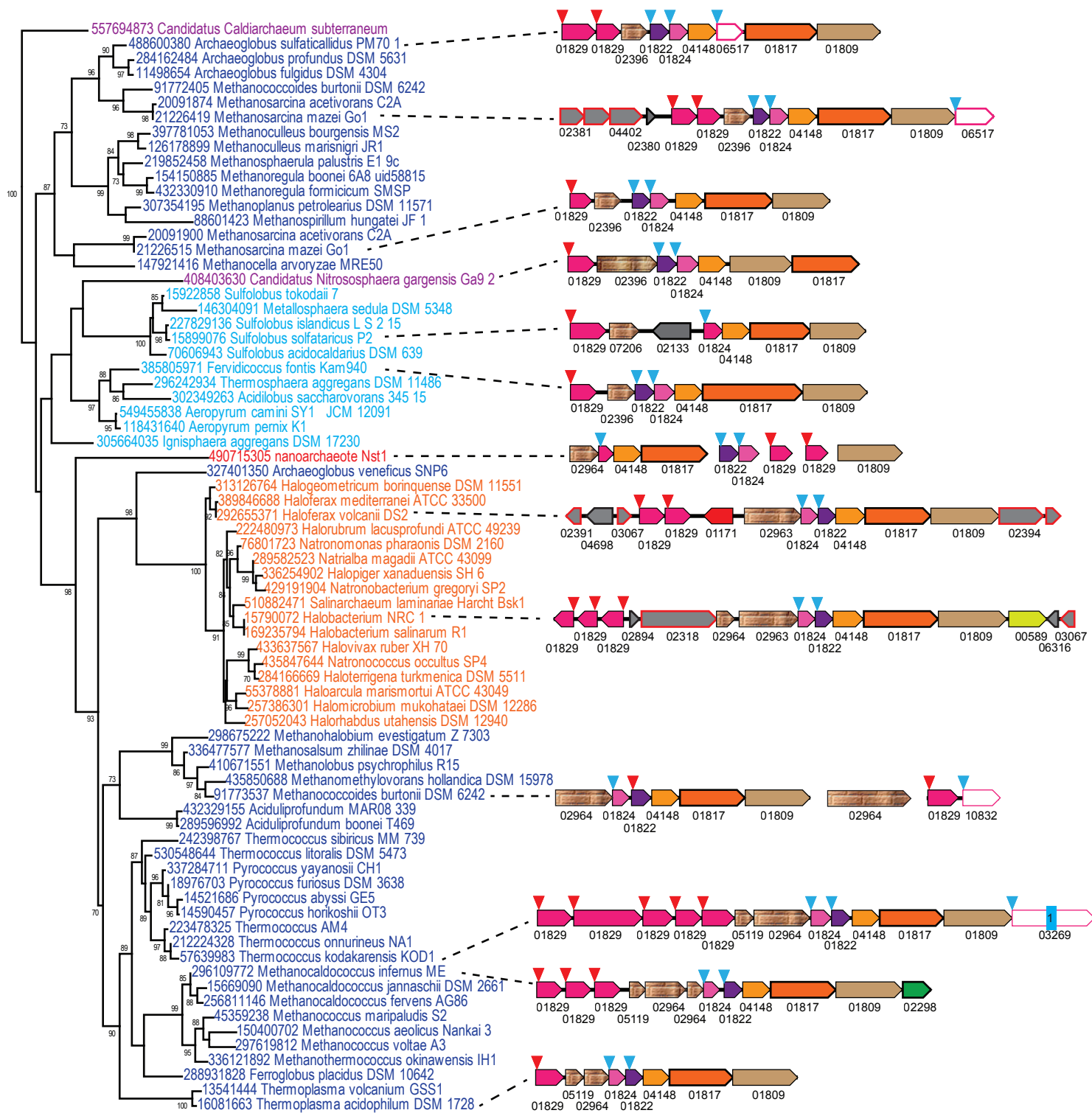

Supplementary Figure S2

Supplement: Supplementary file 1 [file Presentation_1.ZIP › makarova_Frontiers_Figure_S2.pdf]

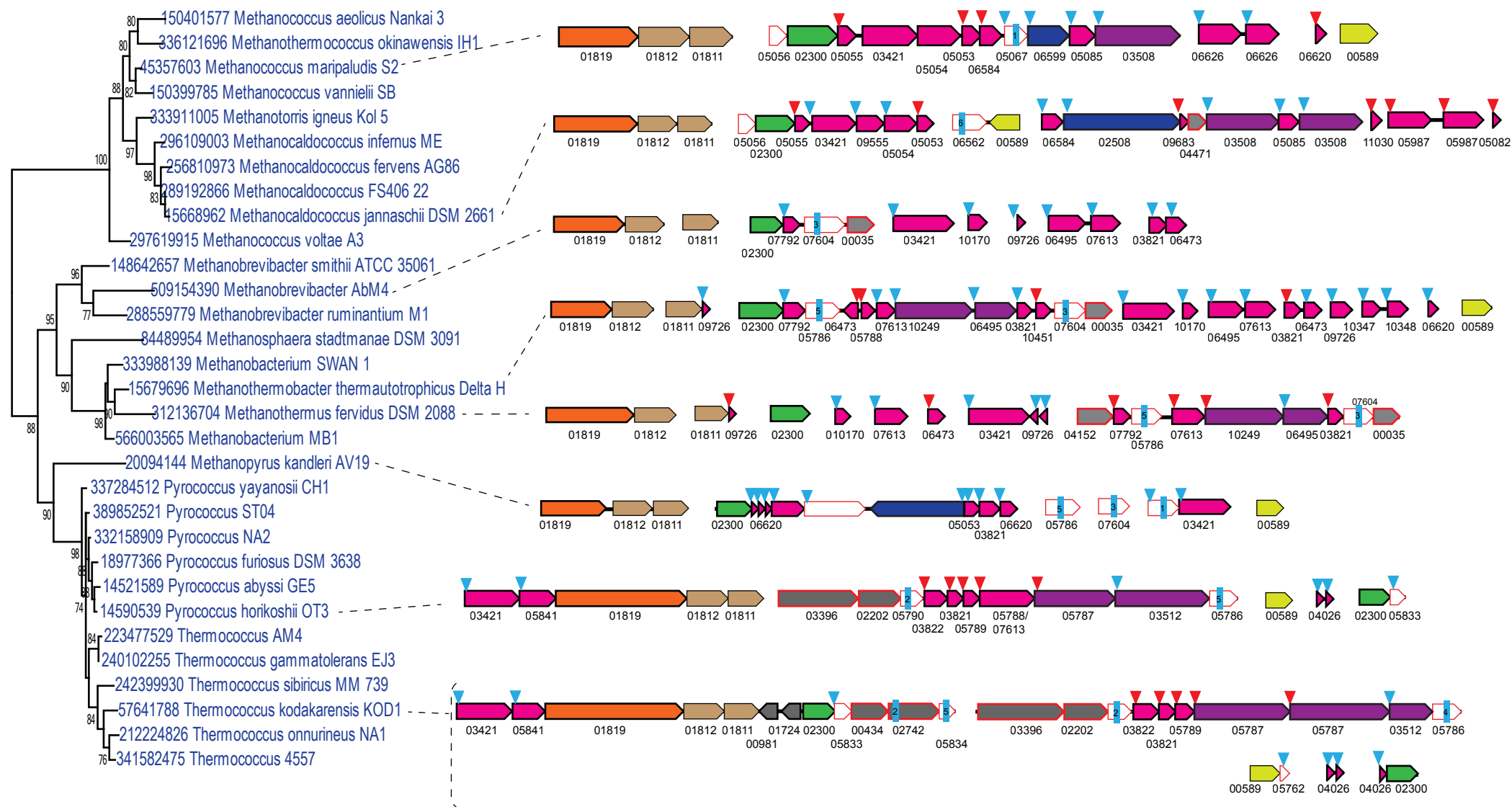

Supplementary Figure S1

Supplement: Supplementary file 1 [file Presentation_1.ZIP › makarova_Frontiers_Figure_S1.pdf]
