# Supplementary material for: Diversity and Evolution of Type IV pili Systems in Archaea
Source: Front Microbiol. 2016 May 6;7:667. doi: 10.3389/fmicb.2016.00667 (PMC4858521; doi:10.3389/fmicb.2016.00667)
Supplement: Supplementary file 1 [file makarova_Frontiers_Figure_S3.pdf › makarova_Frontiers_Figure_S3.pdf]

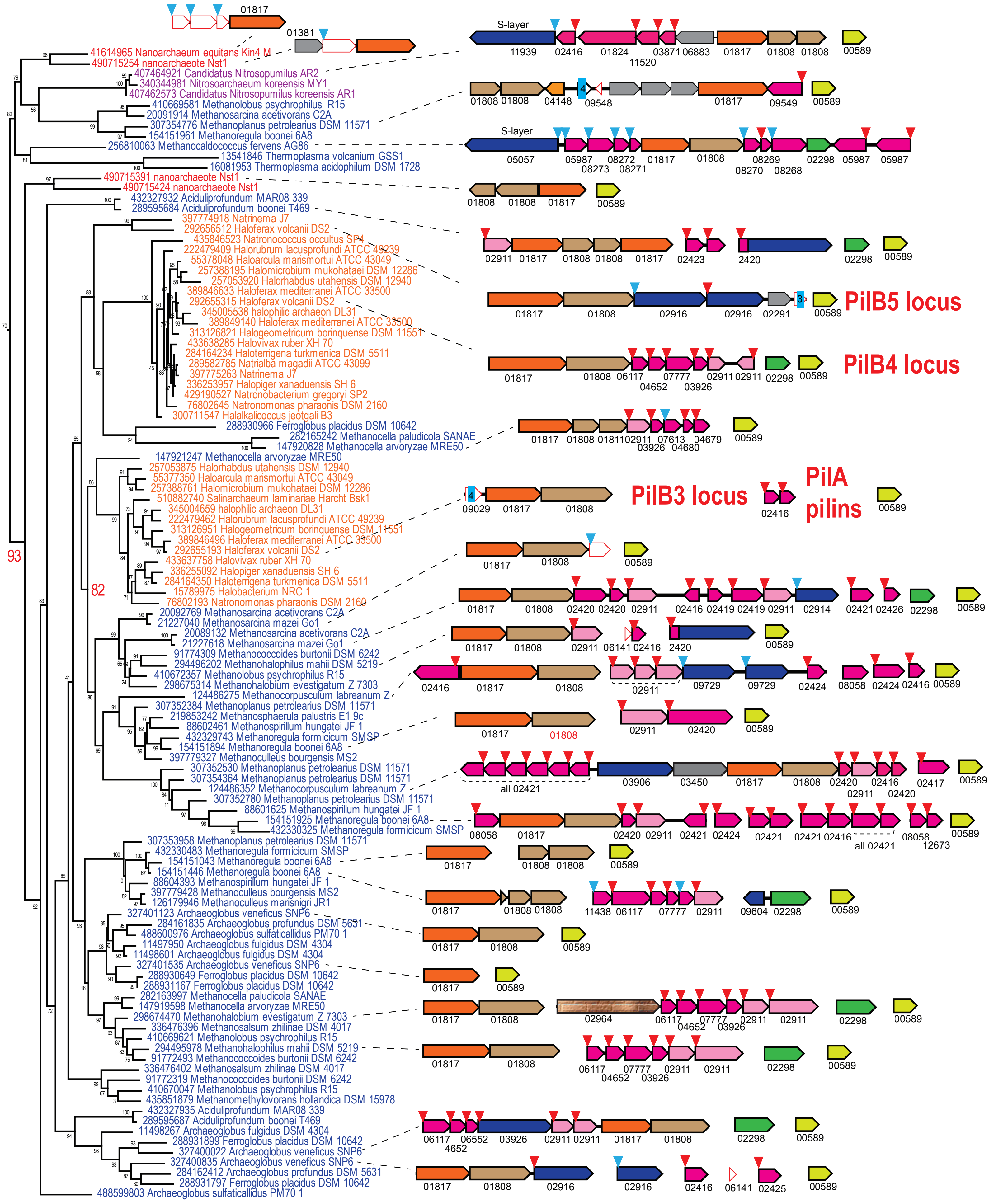

Supplementary Figure S3
